# Circulating small extracellular vesicles mediate vascular hyperpermeability in diabetes

**DOI:** 10.1007/s00125-024-06120-9

**Published:** 2024-03-15

**Authors:** Dakota Gustafson, Peter V. DiStefano, Xue Fan Wang, Ruilin Wu, Siavash Ghaffari, Crizza Ching, Kumaragurubaran Rathnakumar, Faisal Alibhai, Michal Syonov, Jessica Fitzpatrick, Emilie Boudreau, Cori Lau, Natalie Galant, Mansoor Husain, Ren-Ke Li, Warren L. Lee, Rulan S. Parekh, Philippe P. Monnier, Jason E. Fish

**Affiliations:** 1 Toronto General Hospital Research Institute, University Health Network, Toronto, ON Canada; 2 Department of Laboratory Medicine and Pathobiology, University of Toronto, Toronto, ON Canada; 3 Division of Fundamental Neurobiology, Toronto Western Research Institute, University Health Network, Toronto, ON Canada; 4 Keenan Research Centre for Biomedical Science, St Michael’s Hospital, Toronto, ON Canada; 5 Institute of Medical Science, University of Toronto, Toronto, ON Canada; 6 Department of Medicine and Pediatrics, Women’s College Hospital, Hospital for Sick Children and University of Toronto, Toronto, ON Canada; 7 Department of Biochemistry, University of Toronto, Toronto, ON Canada; 8 Donald K. Johnson Eye Institute, Krembil Research Institute, University Health Network, Toronto, ON Canada; 9 Department of Physiology, Faculty of Medicine, University of Toronto, Toronto, ON Canada; 10 Peter Munk Cardiac Centre, University Health Network, Toronto, ON Canada

**Keywords:** Cerebrovasculature, Cognitive impairment, Diabetes, Endothelial dysfunction, Extracellular vesicles, Permeability, Type 2 Diabetes, Vascular biology, Vascular hyperpermeability, Vasculature

## Abstract

**Aims/hypothesis:**

A hallmark chronic complication of type 2 diabetes mellitus is vascular hyperpermeability, which encompasses dysfunction of the cerebrovascular endothelium and the subsequent development of associated cognitive impairment. The present study tested the hypothesis that during type 2 diabetes circulating small extracellular vesicles (sEVs) exhibit phenotypic changes that facilitate pathogenic disruption of the vascular barrier.

**Methods:**

sEVs isolated from the plasma of a mouse model of type 2 diabetes and from diabetic human individuals were characterised for their ability to disrupt the endothelial cell (EC) barrier. The contents of sEVs and their effect on recipient ECs were assessed by proteomics and identified pathways were functionally interrogated with small molecule inhibitors.

**Results:**

Using intravital imaging, we found that diabetic mice (*Lepr*^db/db^) displayed hyperpermeability of the cerebrovasculature. Enhanced vascular leakiness was recapitulated following i.v. injection of sEVs from diabetic mice into non-diabetic recipient mice. Characterisation of circulating sEV populations from the plasma of diabetic mice and humans demonstrated increased quantity and size of sEVs compared with those isolated from non-diabetic counterparts. Functional experiments revealed that sEVs from diabetic mice or humans induced the rapid and sustained disruption of the EC barrier through enhanced paracellular and transcellular leak but did not induce inflammation. Subsequent sEV proteome and recipient EC phospho-proteome analysis suggested that extracellular vesicles (sEVs) from diabetic mice and humans modulate the MAPK/MAPK kinase (MEK) and Rho-associated protein kinase (ROCK) pathways, cell–cell junctions and actin dynamics. This was confirmed experimentally. Treatment of sEVs with proteinase K or pre-treatment of recipient cells with MEK or ROCK inhibitors reduced the hyperpermeability-inducing effects of circulating sEVs in the diabetic state.

**Conclusions/interpretation:**

Diabetes is associated with marked increases in the concentration and size of circulating sEVs. The modulation of sEV-associated proteins under diabetic conditions can induce vascular leak through activation of the MEK/ROCK pathway. These data identify a new paradigm by which diabetes can induce hyperpermeability and dysfunction of the cerebrovasculature and may implicate sEVs in the pathogenesis of cognitive decline during type 2 diabetes.

**Graphical Abstract:**

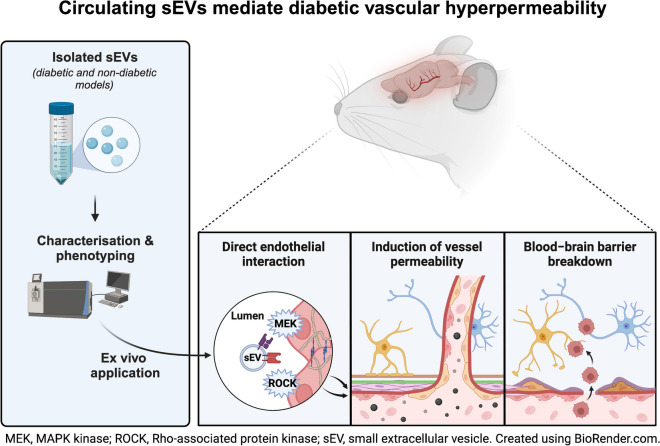

**Supplementary Information:**

The online version of this article (10.1007/s00125-024-06120-9) contains peer-reviewed but unedited supplementary material.


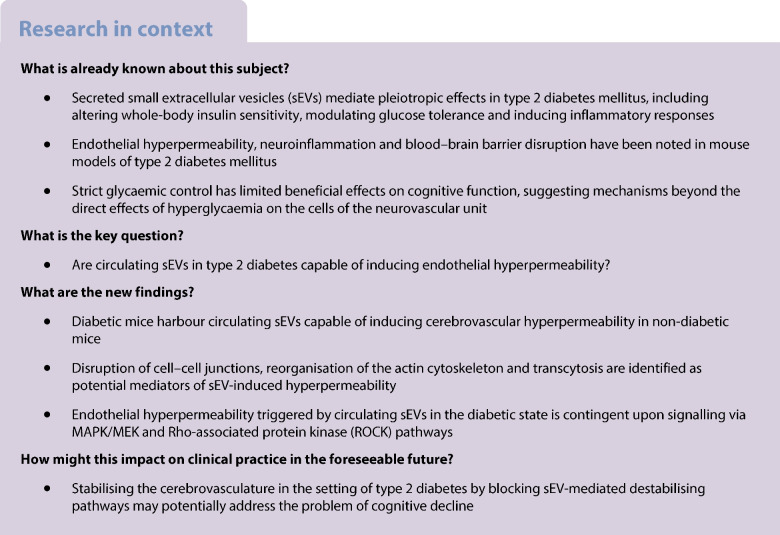


## Introduction

Once considered a simplistic barrier between the blood and the vessel wall, the endothelium is now regarded as a dynamic and complex endocrine organ responsible for maintaining homeostasis across the vascular system [1]. Endothelial cells (ECs) exhibit a diverse array of phenotypes to meet the unique physiological requirements of different tissues [2–4]. This is especially the case in the central nervous system, wherein the functional architecture of the brain is partitioned from the periphery via a dedicated microvascular layer, the blood–brain barrier (BBB) [5]. Relative to the peripheral endothelium, the BBB features a high electrical resistance, lacks fenestrations and exhibits low rates of transcytosis, all of which contribute to the formation of a tightly regulated barrier capable of maintaining parenchymal homeostasis [6].

Dysfunction of the BBB has been implicated in both age- and pathology-related neurodegeneration and is accompanied by reduced cerebral blood flow, loss of tight junction integrity, increases in transcytosis and leakage of neurotoxic factors, which together perturb the homeostatic environment needed for optimal neuronal function [7, 8]. For example, individuals with type 2 diabetes, one of the most prevalent chronic diseases, exhibit widespread endothelial dysfunction, which occurs even at the level of the BBB [9, 10]. Breakdown of cell-to-cell junctions, initiation of local proinflammatory responses and induction of tissue hyperpermeability are hallmarks of well-studied microvascular complications, including diabetic retinopathy, neuropathy and oedema, all of which remain principal drivers of morbidity [11]. These features have also been noted in mouse models of type 2 diabetes, including leptin-receptor-deficient mice (*Lepr*^db/db^) [12–15].

Despite recognition that individuals with type 2 diabetes are at increased risk for cognitive impairment, dementia and stroke, there remains a significant gap in our understanding of the preceding microvascular disruption at the level of the BBB that underlies these conditions [16]. Notably, strict glycaemic control provides limited beneficial effects on cognitive function, suggesting mechanisms beyond the direct detrimental effects of hyperglycaemia on the cells of the neurovascular unit [17, 18]. To this end, extracellular vesicles (EVs), a heterogeneous group of small membranous protein-harbouring and RNA-containing structures, have garnered increasing attention as dynamic inter-organ communicators of cellular homeostasis [19, 20]. Vesicles secreted from various cell types have systemic capabilities in vivo, including the ability to alter whole-body insulin sensitivity, modulate glucose tolerance and induce inflammatory responses, which have collectively unveiled new avenues for understanding the pleiotropic effects of type 2 diabetes [21–23]. Here, we used both ex vivo and in vivo modelling to determine the mechanisms whereby circulating small EVs (sEVs) are modulated during type 2 diabetes and subsequently uncover how they disrupt the vascular barrier.

## Methods

Details of materials and methods can be found in the Electronic Supplementary Material (ESM) Methods and ESM Table 1.

### Human study participants

All participants were 18 years of age or older and provided a blood sample after written informed consent under protocols approved by the Johns Hopkins University research ethics board (REB no. NA00012150) as part of the Family Investigation of Nephropathy and Diabetes study (ClinicalTrials.gov registration no. NCT00301249). Subsequent research was conducted under protocols approved by SickKids Hospital (REB no. 1000047532) as well as University Health Network (REB no. 16-6229.0), with all associated protocols conforming to the ethical principles for medical research set forth by the Helsinki Declaration II [24]. As described previously in a case–control design [25], participants were defined as cases (i.e. diabetic) if they had diabetic nephropathy with type 2 diabetes and were currently or previously treated with insulin, oral glucose-lowering agents, or both, and also if HbA_1c_ values were ≥42 mmol/mol (6.0%) [26]. In contrast, participants were defined as controls (i.e. non-diabetic) if they showed no evidence of diabetic nephropathy, diabetes, known CVD (i.e. history of congestive heart failure, myocardial infarction, transient ischaemic attack, high cholesterol, coronary artery bypass, angioplasty, hypertension and/or chronic obstructive pulmonary disease) or cancer. An equal number of diabetic and non-diabetic participants were recruited, with participants additionally being matched for age and sex (ESM Methods). There were 15 participants in each group (80% male; self-reported) with a mean ± SD age of 43.29±9.14 years in the diabetic group and 43.21±10.09 years in the non-diabetic group.

### Murine models

All in vivo procedures were approved by the University Health Network Animal Ethics Committee (Animal Utilisation Protocol no. 2291.23), and animals received humane care in line with the ‘Guide for the care and use of laboratory animals’ (National Research Council, 8th Edition, revised 2011) as well as in accordance with the Canadian Council on Animal Care Guidelines [27, 28]. Healthy male mice, heterozygous for *Dock7*^m^ and heterozygous for *Lepr*^db^ (*BKS.Cg-Dock7*^m/+^ +/+ *Lepr*^db^*/J*; referred to as *db/+*) or homozygous for *Lepr*^db^ (also known as *db/db*), were procured from Jackson Research Laboratory (Bar Harbour, MI, USA). Only male mice were used in this study since male mice more effectively recapitulate the vascular effects of type 2 diabetes [29, 30]. Mice of each genotype (maximum five per cage) were housed together under standard conditions (12 h light–dark cycle, 22–25°C) in vented micro-isolator cages, with free access to standard chow (rodent diet with 10% of energy from fat; catalogue no. D12450B, Research Diets, New Brunswick, NJ, USA) and water. No mice were removed from the study and there were no adverse outcomes. Details of intravital microscopy can be found in the ESM Methods.

### Enrichment, analysis and quality control of circulating sEVs from plasma

#### Human

Enrichment for sEVs from human plasma was conducted by size-exclusion chromatography (SEC) using first-generation qEV single (70 nm) columns (Izon Science, Medford, MA, USA) by combining eluate fractions six through ten; following the MIBlood-EV reporting framework (ESM Methods) [31]. Briefly, 1 ml bed volume qEV columns were pre-cleared with fresh filtered 0.22 µm filtered Dulbecco’s PBS without Ca^2+^ or Mg^2+^ (PBS^−/−^; Sigma-Aldrich, St Louis, MO, USA) and 150 µl of platelet-poor plasma was loaded onto the qEV upper frit. sEVs were eluted with 0.22 µm filtered PBS^−/−^ with the first 1 ml of eluate being considered the void volume and the following five 200 µl fractions considered sEV-enriched factions. Fractions six through ten were concentrated using an Amicon Ultra-15 Centrifugal Filter Unit (MilliporeSigma, Burlington MA, USA) with a 10,000 Dalton molecular weight cut-off filter (4000 *g*, 4°C, 40 min); final volumes were ~100–200 µl. The protein concentration of the fractions was measured using a BCA Protein Assay Kit for Low Concentration (Abcam, Cambridge, MA, USA) using a Cytation5 Imaging Reader (BioTek, Winooski, VT, USA). Protein concentrations were subsequently interpolated using a non-linear fit of BSA standards.

#### Murine

sEVs were collected from mice in an identical manner, ensuring the isolation of similar fractions. Additional analyses were conducted on complete sEV populations from 150 μl of mouse plasma following cardiac puncture using ExoQuick (System Biosciences, Palo Alto, CA, USA) according to the manufacturer’s instructions (as before [23]); sEVs were resuspended in 100 μl of PBS^−/−^ (see ESM Methods for additional details regarding analysis and quality control of vesicle preparations and ESM Table 2 for settings used for nanoparticle tracking analysis [NTA]).

### Biochemistry, gene expression and cell biology experiments

For biochemistry, gene expression and cell biology experiments, sEVs were used at a concentration of ~0.5–1.0×10^10^ particles/ml, which approximates published physiological ranges [32]. See ESM Methods for details. Primer sequences for quantitative RT-PCR experiments are provided in ESM Table 3.

### Statistical analysis and data visualisation

The normality of data distribution was evaluated using the Shapiro–Wilk test. If distributions were normal, we used unpaired Student’s *t* tests for two-group comparisons and one-way ANOVA analysis with a Tukey’s or Bonferroni post hoc test (where appropriate) when more than two groups were compared. If distributions were non-normal, we used the Mann–Whitney *U* test for the analysis of two groups and the Kruskal–Wallis test with Dunn’s post hoc test for multiple-group comparisons. A *p*<0.05 threshold was considered statistically significant and indicated in the graphs as reported by the analysis software with significance thresholds of *p*<0.05, *p*<0.01 and *p*<0.001. No datapoints were excluded from analysis. Data were analysed with GraphPad Prism 9.0.0 for MacOS (GraphPad Software, La Jolla, CA, USA), schematics were created with BioRender.com, and final figures assembled for publication purposes using Adobe Illustrator (v27.8.1). Fiji-ImageJ was utilised to quantify the in vivo multiphoton microscopic imaging and leakage quantification (https://fiji.sc/), accessed 12 March 2019. Direct links to the utilised software are available in ESM Table 1. Details on randomisation and blinding are provided in ESM Methods.

## Results

### Characterisation of plasma sEVs from diabetic mice and humans

**Fig. 1:**
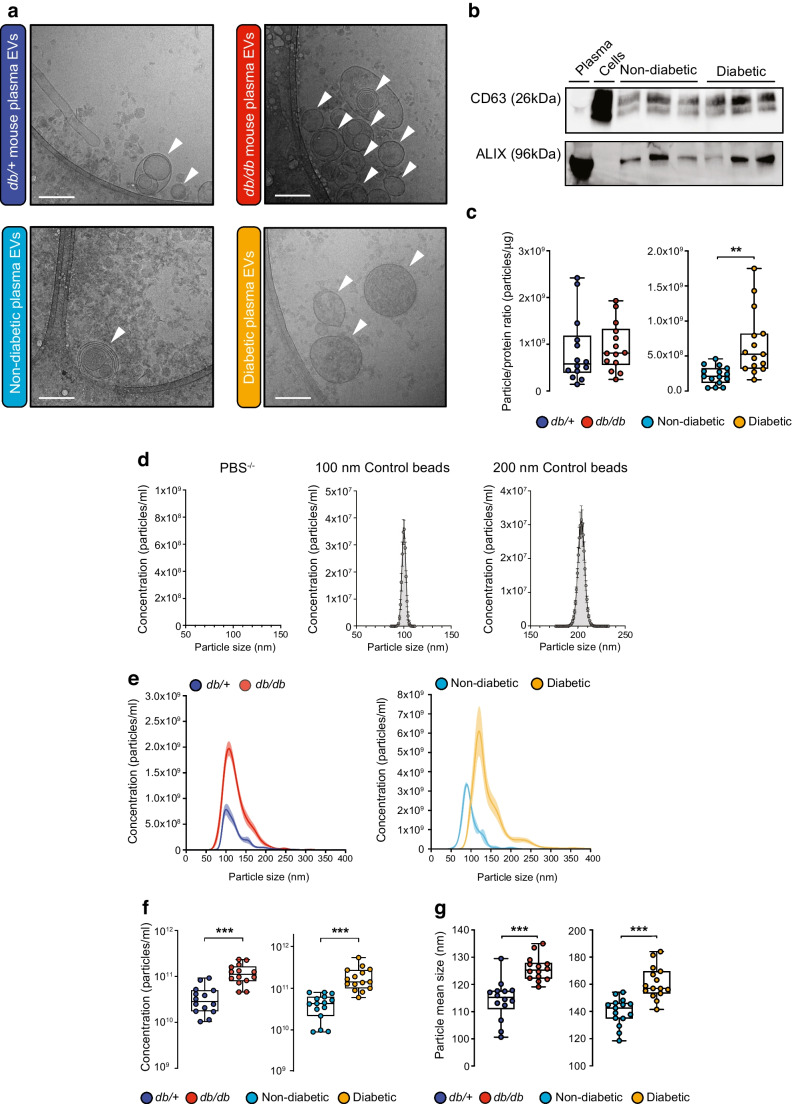
Diabetes increases the size and concentration of plasma sEVs. (**a**) Cryo-EM images of EVs isolated by SEC from the plasma of *db/db* and *db/*+ mice as well as diabetic and non-diabetic participants. A representative image is shown, with arrowheads indicating lipid-bilayer morphology consistent with sEV structure. Scale bar, 200 nm. (**b**) Canonical sEV markers CD63 and ALIX were assessed by immunoblot in SEC-enriched samples from non-diabetic and diabetic human plasma; *n*=3 biological replicates per group are shown. (**c**) Quantification of particles/µg of protein from murine (*p*=0.72, *n*=14 biological replicates) and human (*p*=0.0012, *n*=15 biological replicates) sEVs isolated from plasma using SEC. High particle concentrations compared with protein concentration are indicative of pure sEV samples. (**d**) Validation of NTA using SEC-eluted (mock-treated) filtered PBS^−/−^, 100 nm control polystyrene beads, and 200 nm polystyrene beads. Values are presented as the mean ± SD (*n*=3 technical replicates). (**e**) NTA traces of aggregated sEV-enriched fractions (*n*=3 biological replicates per group) from mice or humans. (**f**) Quantification of the concentration of sEVs from murine (*p*<0.001, *n*=14 biological replicates) and human (*p*<0.001, *n*=15 replicates) plasma. (**g**) Quantification of the mean particle size of sEVs from murine (*p*<0.001, *n*=14 biological replicates) and human (*p*<0.001, *n*=15 replicates) plasma. (**c**, **f**, **g**) Boxes depict the median with upper and lower quartiles and the whiskers depict the minimum and maximum. The data were analysed using the Mann–Whitney test; (**p<0.01, ***p<0.001)

Initial characterisation focused on examining the impact of type 2 diabetes on the phenotype of circulating sEVs in both a murine model and human study participants. Mice homozygous for a mutant allele of the leptin receptor (i.e. *BKS-Lepr*^db^*/J* mice [*Lepr*^db^*/J*]; denoted as *db/db*) were assessed, given their ability to recapitulate key features of type 2 diabetes (ESM Fig. 1) [23]. Circulating sEVs were assessed when mice were aged 14 weeks, a stage characterised by microvascular defects in both the heart and brain; overt cardiac dysfunction and cognitive impairment occur latently (i.e. ~26 weeks) [15, 23]. Isolation of sEVs from the plasma of 14-week-old diabetic *db/db* or non-diabetic *db/*+ control mice was facilitated using both precipitation (i.e. ExoQuick) and size-exclusion chromatography (SEC); human plasma sEV samples from diabetic and non-diabetic individuals were exclusively isolated by SEC. Supplemental quality control analyses of sEVs isolated by ExoQuick from this mouse model at this time point, including western blotting, cryogenic electron microscopy (cryo-EM) and NTA have been published elsewhere [23]. NTA and protein quantification analysis of SEC-enriched sEVs from mouse and human plasma revealed that fractions six through ten contained the highest concentration of sEVs while minimising the co-isolation of plasma proteins (ESM Fig. 2). To further demonstrate the purity of isolated vesicles, cryo-EM was performed, revealing characteristic sEV morphologies (Fig. 1a and ESM Fig. 3a, b). Western blotting revealed that the EV preparations contained the classic EV markers CD63 and ALG-2-interacting protein X (ALIX; Fig. 1b). High particle to protein ratios were also suggestive of the high purity of EV preparations (Fig. 1c). Standardised clinical chemistry profiling was also run to assess the co-purification of lipoproteins (e.g. total cholesterol, triglycerides, HDL-cholesterol, LDL-cholesterol) and glucose with isolated sEV samples (ESM Fig. 3c). This analysis revealed that sEVs isolated from plasma by SEC do not contain high levels of lipoprotein contaminants or glucose, both of which could confound EV functional experiments. Finally, NTA demonstrated that plasma sEVs from diabetic mice or diabetic humans were both larger and more abundant than their non-diabetic counterparts (Fig. 1d–g).

### Plasma sEVs from diabetic mice and humans induce EC permeability in vitro

**Fig. 2:**
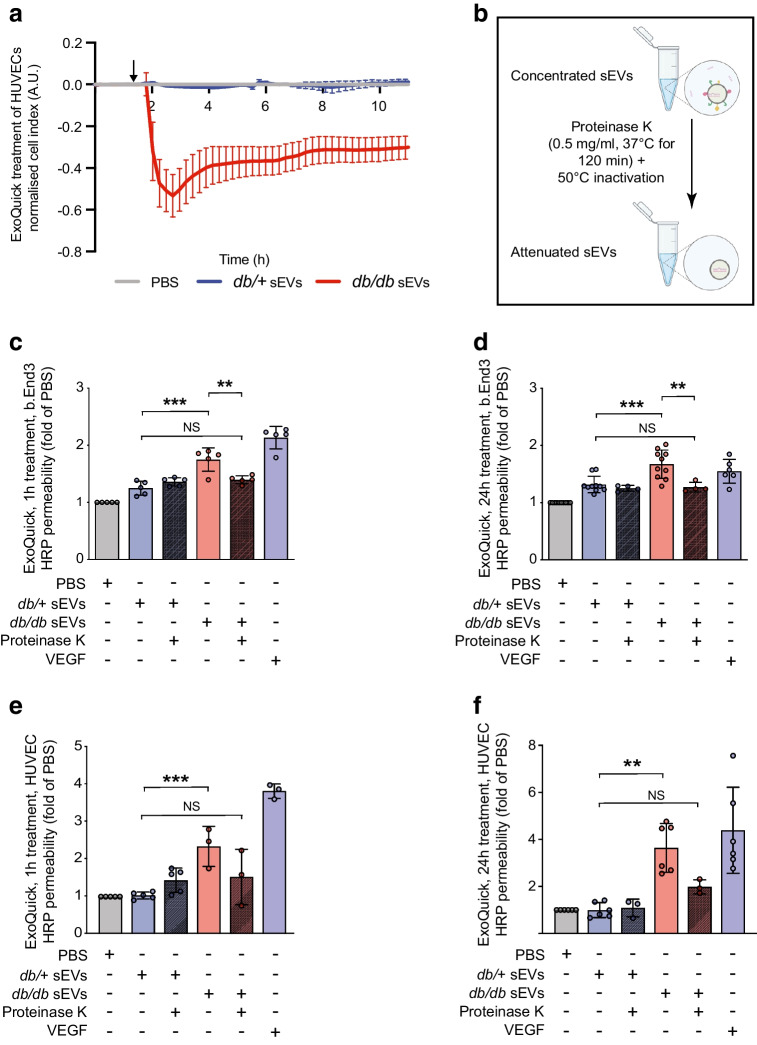
sEVs isolated by ExoQuick from diabetic mouse plasma induce rapid and sustained increases in endothelial permeability, dependent on vesicle surface proteins. (**a**) Traces of normalised cell index using xCELLigence-based impedance measurements in confluent HUVEC monolayers treated with PBS^−/−^ (set to 0) or sEVs isolated by ExoQuick from an equal volume of either *db/*+ (*n*=3) or *db/db* (*n*=3) mouse plasma; arrow indicates the time point when sEVs were added. (**b**) Schematic showing the proteinase K treatment protocol of total vesicular precipitates (created with BioRender.com). (**c**) A transwell permeability assay was performed in b.End3 brain ECs exposed for 1 h (acute) to PBS or ExoQuick-isolated sEVs (*db/*+ or *db/db*) isolated from an equal volume of plasma; VEGF was added for 30 min as a positive control. (**d**) Permeability in b.End3 cells treated for 24 h (chronic) with ExoQuick-isolated sEVs (*db/*+ or *db/db*) from an equal volume of plasma with or without proteinase K pre-treatment. (**e**) Permeability in HUVECs following a 1 h treatment (acute) with ExoQuick-isolated sEVs (*db/*+ or *db/db*) from an equal volume of plasma with or without proteinase K pre-treatment. (**f**) Permeability in HUVECs following 24 h treatment (chronic) with ExoQuick-isolated sEVs (*db/*+ or *db/db*) from an equal volume of plasma with or without proteinase K pre-treatment. The number of biological replicates is indicated in the figure at the bottom of each bar in (**c**–**f**). For (**c**–**f**) data are relative to the PBS control and are displayed as fold of PBS. The data were analysed using ANOVA with Tukey’s post hoc test; **p<0.01, ***p<0.001. Values are presented as the mean ± SD. A.U., arbitrary units

To determine the impact of diabetic sEVs on EC permeability, we used xCELLigence Real-Time Cell Analysis, which measures impedance as a proxy of cell barrier integrity. Exposure of a confluent monolayer of HUVECs to ExoQuick-isolated sEVs from 14-week-old *db/db* mice (~10^10^ particles/ml) led to rapid loss of impedance within 15–30 min, sustained for >8 h (Fig. 2a). In contrast, sEVs isolated from the same volume of non-diabetic (*db/+*) mouse plasma did not disrupt the barrier. We next sought to determine whether circulating sEVs from *db/db* mice could disrupt the barrier of the mouse brain EC line, b.End3. Cells were plated on transwell inserts with 3 μm pore size and grown to confluence; following exposure to sEVs for 1 h or 24 h, barrier function was assessed using the transit of horseradish peroxidase (HRP) as a surrogate (Fig. 2c,d). sEVs isolated from *db/db* mice increased permeability to HRP at both of these time points, similar to vascular endothelial growth factor (VEGF) stimulation (used as a positive control of a vascular permeabilising factor), while sEVs from *db/+* mice did not (Fig. 2c,d). Similarly, *db/db* mouse sEVs could increase HRP permeability following 1 h or 24 h treatment of HUVECs (Fig. 2e,f). Removal of surface proteins by proteinase K pre-treatment of the sEVs negated the barrier-disrupting properties of *db/db* sEVs (Fig. 2b–f).

**Fig. 3:**
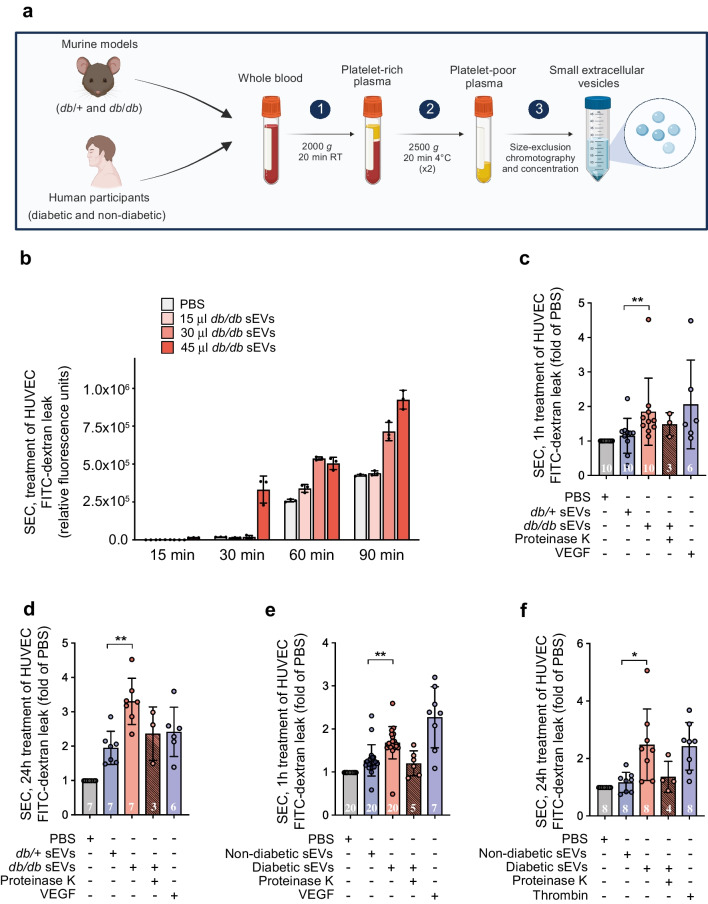
sEVs isolated by SEC from diabetic mouse or human plasma induce rapid and sustained increases in endothelial permeability. (**a**) Schematic detailing the SEC protocol for the enrichment of sEVs from murine and human plasma (see ESM Figs 2, 3 for further details and quality control metrics) (created with BioRender.com). (**b**) Kinetics of transwell permeability in HUVEC confluent monolayers treated with various amounts of SEC-isolated *db/db* plasma sEVs for the specified periods, demonstrating that leakage occurs rapidly following treatment in a concentration-dependent manner (*n*=3 biological replicates). FITC–dextran levels are indicated as relative fluorescence units. (**c**) Permeability in HUVECs following 1 h treatment (acute) with SEC-isolated sEVs (*db/*+ or *db/db* mice) from an equal volume of plasma. (**d**) Permeability in HUVECs following 24 h treatment (chronic) with SEC-isolated sEVs (*db/*+ or *db/db* mice) from an equal volume of plasma. (**e**) Permeability in HUVECs following 1 h treatment (acute) with SEC-isolated sEVs (diabetic or non-diabetic humans) from an equal volume of plasma. (**f**) Permeability in HUVECs following 24 h treatment (chronic) with SEC-isolated sEVs (diabetic or non-diabetic humans) from an equal volume of plasma. VEGF was used as a positive control in panels (**c**–**e**), and thrombin was used as a positive control in panel (**f**). In (**c**–**f**), the number of biological replicates is indicated at the bottom of each bar; data are relative to the PBS control and are displayed as fold of PBS. The data were analysed using ANOVA with Tukey’s post hoc test *p<0.05, **p<0.01. Values are presented as the mean ± SD. RT, room temperature

We next determined whether sEVs isolated by an independent method would have similar properties by exposing HUVEC monolayers to SEC-enriched sEVs from equal volumes of diabetic or non-diabetic mouse or human plasma (Fig. 3). To better understand the kinetics and dose-dependence of sEV-mediated permeability in diabetes, we first assessed permeability at 15, 30, 60 and 90 min to FITC–dextran with increasing doses of *db/db* sEVs. Analysis revealed that the highest dose of diabetic sEVs could induce leakage as early as 30 min and that there was a dose- and time-dependent increase in permeability (Fig. 3b). Using the highest dose of sEVs (~10^10^ particles/ml), we found that both SEC-isolated *db/db* mouse sEVs (Fig. 3c,d) and diabetic human sEVs (Fig. 3e,f) could induce significant leakage after 1 h or 24 h of exposure compared with non-diabetic mouse and human sEVs (isolated from an equal volume of plasma); an increase in permeability of similar magnitude to VEGF stimulation. Treatment of isolated sEVs with proteinase K again abrogated their ability to induce hyperpermeability (Fig. 3c–f). We also performed experiments in which an equal number of sEVs from *db/db* or *db/+* mouse plasma were added to HUVECs, and this revealed increased leakage from diabetic sEV treatment (ESM Fig. 4). Taken together, these data demonstrate that sEVs isolated from the plasma of mice or humans with diabetes can induce rapid and sustained EC permeability at physiologically relevant doses.

### Cerebrovasculature of diabetic mice exhibits enhanced permeability

**Fig. 4:**
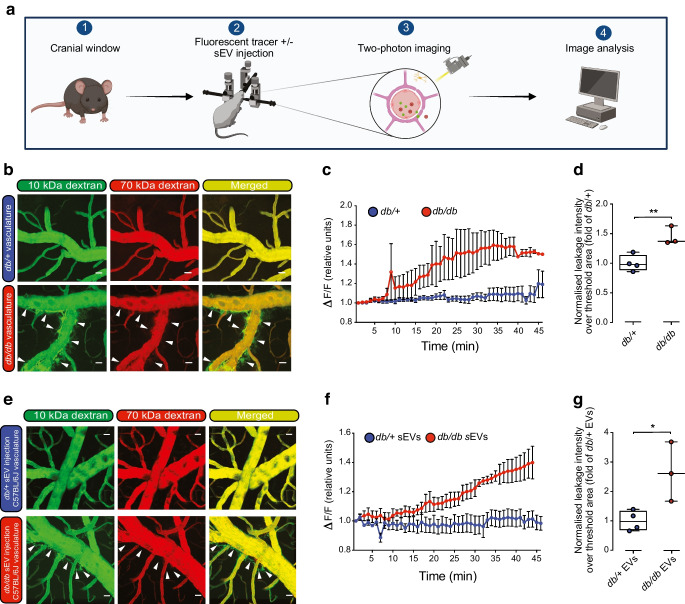
sEVs from diabetic mice induce cerebrovascular permeability in vivo. (**a**) Schematic detailing the two-photon intravital microscopy protocol utilised to examine cerebrovascular permeability (created with BioRender.com). (**b**) Representative still images from in vivo multiphoton intravital microscopy videos following retro-orbital injection of 70 kDa Texas Red–dextran and 10 kDa FITC–dextran into 14-week-old *db/*+ or *db/db* mice (BKS background) 45 min after injection. Arrowheads indicate regions of leakage of the 10 kDa tracer. Scale bar, 25 μm. *db/*+, *n*=4 biological replicates; *db/db*, *n*=3 biological replicates. (**c**) Assessment of 10 kDa FITC–dextran and 70 kDa Texas Red–dextran leakage over a 45 min period in *db/+* and *db/db* mice with the change in fluorescence quantified over the observed region of interest (ΔF/F). Time zero was set to 1 for each group. (**d**) Total normalised leakage intensity over a 45 min time period quantified over total threshold area in *db/+* and *db/db* mice. Data are plotted as fold of *db/+.* **p<0.01 by unpaired *t* test. (**e**) Representative still images from in vivo multiphoton intravital microscopy videos following retro-orbital injection of 70 kDa Texas Red–dextran and 10 kDa FITC–dextran into wild-type C57Bl/6J mice that were injected retro-orbitally with *db/*+ or *db/db* sEVs isolated from an equal volume of plasma (after 45 min). Scale bar, 25 μm. *db/*+ sEVs, *n*=4 biological replicates; *db/db* sEVs, *n*=3 biological replicates. (**f**) Assessment of 10 kDa FITC–dextran and 70 kDa Texas Red–dextran leakage over a 45 min period immediately after sEV injection with the change in fluorescence quantified over the observed region of interest (ΔF/F). Time zero was set to 1 for each group. (**g**) Total normalised leakage intensity over a 45 min period quantified over the total threshold area in mice treated with *db/+* and *db/db* EVs. Data are presented as fold of *db/+* EVs. *p<0.05 by unpaired *t* test. (**d**, **g**) Boxes depict the median with upper and lower quartiles and the whiskers depict the minimum and maximum

To assess the integrity of blood vessels in the brain in this mouse model of type 2 diabetes, we performed intravital microscopy to measure the leakage of fluorescent dye out of the vasculature (Fig. 4a). Two dyes were simultaneously injected via retro-orbital injection: a larger Texas-Red-labelled 70 kDa dextran; and a smaller FITC-labelled 10 kDa dextran (1:1). The larger dye only leaks out of vessels that are disrupted due to haemorrhage, while the smaller dye can leave the vessel due to changes in EC permeability, mediated by transcellular and/or paracellular leakage. Assessment over a 45 min period revealed extensive leakage of the 10 kDa dextran from the brain vasculature of *db/db* mice but not non-diabetic *db/*+ mice (Fig. 4b–d). The 70 kDa tracer demonstrated only minor amounts of leakage out of the vasculature in *db/db* mice, suggesting the leakiness of blood vessels is due to increased permeability.

### sEVs from diabetic mice induce cerebrovascular leakage in non-diabetic mice

To determine whether circulating sEVs might contribute to the enhanced cerebrovascular leakiness in type 2 diabetes, we enriched sEVs by SEC from an equal volume of plasma from 14-week-old *db/db* or *db*/+ mice. sEVs (150 μl, representing ~1.5×10^10^ sEV particles for *db/db* mice and ~0.75×10^10^ sEV particles for *db/+* mice) were injected retro-orbitally into non-diabetic C57Bl/6J mice and cerebrovascular leakage was measured as above using intravital microscopy immediately after injection. Extensive leakage of the 10 kDa but not the 70 kDa tracer was observed over a 45 min time window in mice injected with sEVs isolated from diabetic *db/db* mice but not non-diabetic *db/+* mice (Fig. 4e–g), suggesting that a component of the cerebrovascular permeability is mediated by circulating sEVs.

### Proteomics reveals that sEVs isolated from diabetic mice and humans are predicted to alter cell–cell junctions and remodel the actin cytoskeleton

**Fig. 5:**
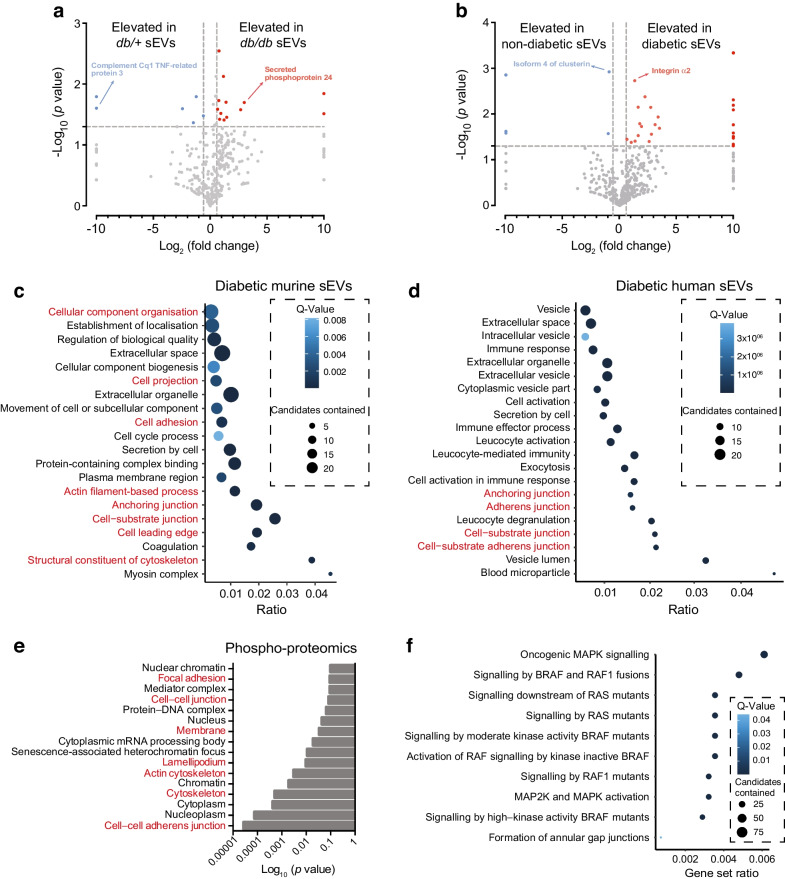
In the diabetic state sEVs are enriched in proteins predicted to modulate pathways involved in cell–cell junctions and cytoskeletal dynamics and activate MAPK and RAS/Rho-GTPase pathways. (**a**, **b**) Volcano plot representations of sEV proteins regulated by diabetic phenotypes from either murine (**a**) or human (**b**) plasma. Blue squares represent proteins that are significantly downregulated and red squares represent proteins that are upregulated by the respective conditions (*n*=3 biological replicates, *p*<0.05). (**c**, **d**) Top 20 enriched Gene Ontology terms from proteins upregulated by diabetes in murine (**c**) and human (**d**) sEVs. The *x*-axis shows the ratio of genes in the category that are differentially expressed with a false-discovery rate <0.05 (enrichment of functions related to the actin cytoskeleton and junctions are highlighted in red). (**e**) Phospho-proteomics was performed on HUVECs treated with sEVs isolated from an equal volume of plasma from non-diabetic or diabetic participants for 1 h to assess the relevancy of identified sEV-enriched proteins. Gene Ontology analysis revealed an enrichment of functional changes related to the actin cytoskeleton and junctions (highlighted in red). (**f**) Signalling pathway analysis of phospho-proteomics data revealed enrichment of signalling pathways linked to RAS/MAPK and ROCK signalling through Rho GTPases. BRAF, B-Raf proto-oncogene, serine/threonine kinase; RAF, rapidly accelerated fibrosarcoma; RAS, rat sarcoma virus

To determine how the protein content of sEVs is impacted by diabetes, we performed tandem MS proteomic analysis of murine and human sEVs isolated from mice/individuals in the diabetic and non-diabetic states. A number of differentially expressed proteins (*p*≤0.05; fold change ≥1.5) were identified in both the murine model and human participants (Fig. 5a,b and ESM Tables 4, 5). Pathway over-representation analysis was conducted on the differentially expressed proteins and revealed several Gene Ontology terms associated with vesicular function as well as signal transduction, actin processes and junctional organisation (Fig. 5c,d). To confirm cellular pathways that might contribute to barrier disruption by diabetic sEVs, phospho-proteomics was performed on HUVECs treated for 1 h with sEVs isolated from an equal volume of plasma from either non-diabetic or diabetic individuals. Analysis revealed that the proteins that were differentially phosphorylated in response to exposure to sEVs isolated from diabetic vs non-diabetic individuals were enriched in gene annotations related to cell–cell junctions and actin cytoskeleton (Gene Ontology terms: focal adhesion, cell–cell junction, membrane, lamellipodium, actin cytoskeleton, cell–cell adherens junctions) (Fig. 5e). Reactome signalling pathway analysis further suggested that alterations to rat sarcoma (RAS)/MAPK signalling, as well as Rho-associated kinase (ROCK) signalling via GTPase modulation [33, 34], may contribute to the observed changes to the EC barrier (Fig. 5f).

### sEVs from mice and humans in the diabetic state do not induce inflammatory signalling but alter actin dynamics, promote disruption of cell–cell junctions and enhance transcytosis

We next sought to understand the impact of sEVs in diabetes at the cellular level. Since diabetes is associated with enhanced inflammation [35], we tested whether sEVs from diabetic individuals activate proinflammatory pathways to induce leakage. While lipopolysaccharide stimulation robustly induced inflammatory markers, sEVs isolated from the plasma of diabetic or non-diabetic individuals had no effect (ESM Fig. 5a). Treatment with sEVs isolated from plasma of *db/+* or *db/db* mice also failed to induce inflammatory genes in treated HUVECs (ESM Fig. 5b). As NF-κB is the principal inflammatory transcriptional pathway, we performed NF-κB reporter assays in hCMEC/D3 cells (a brain microvascular EC line). This revealed no change in response to treatment with sEVs from plasma of diabetic or non-diabetic individuals (ESM Fig. 5c). Finally, immunostaining revealed that sEV treatment did not drive NF-κB (p65) nuclear localisation in HUVECs (ESM Fig. 5d). Thus, EC activation does not appear to be involved in the enhanced permeability in response to exposure to sEVs from diabetic individuals.

**Fig. 6:**
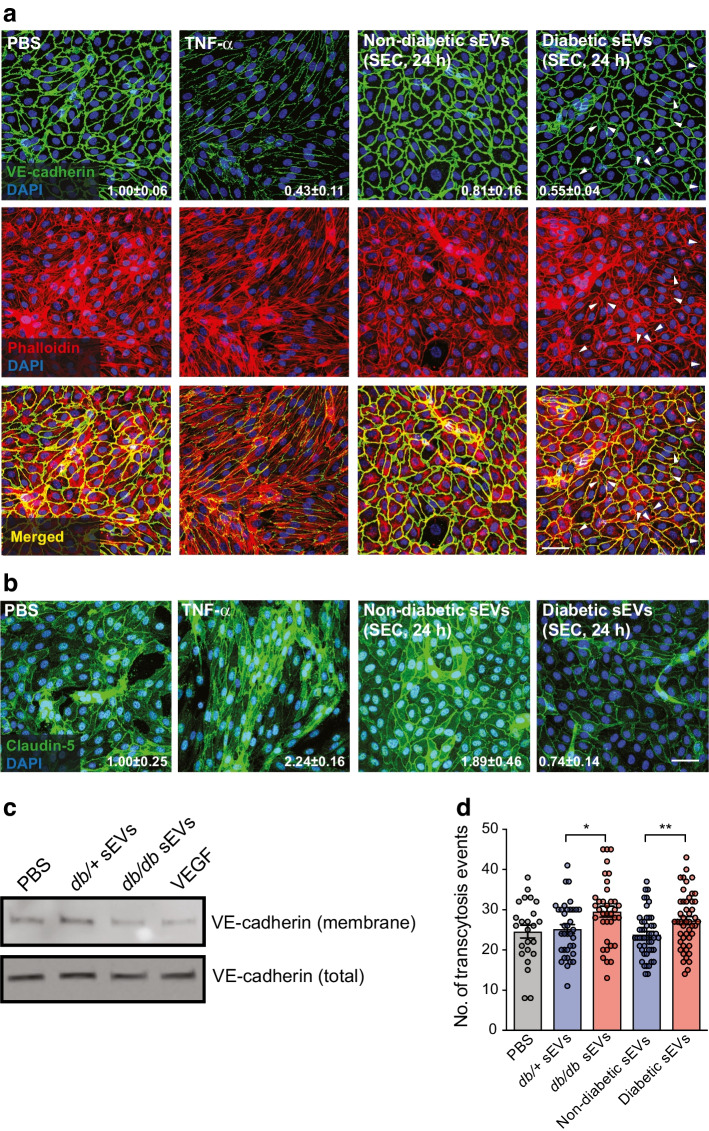
sEVs from diabetic mice or humans induce paracellular and transcellular leakage. (**a**) Adherens junctions (VE-cadherin) and the actin cytoskeleton (phalloidin) were visualised in HUVECs treated for 24 h with PBS or sEVs isolated from an equal volume of plasma from non-diabetic or diabetic individuals. An overlay is shown below. TNF-α treatment was included as a positive control for junctional rearrangement. Less VE-cadherin staining was observed in cells treated with sEVs from diabetic individuals. Arrowheads indicate regions of punctate or missing VE-cadherin staining corresponding to diminished phalloidin staining. A representative experiment is shown (see ESM Fig. 6b for VE-cadherin staining with mouse plasma sEVs). Scale bar, 50 μm. Numbers indicate the mean ± SD of VE-cadherin quantification across three replicate experiments. (**b**) Tight junctions (claudin-5) were visualised in HUVECs treated for 24 h with PBS or sEVs isolated from an equal volume of plasma from non-diabetic or diabetic individuals. TNF-α treatment was included as a positive control for junctional rearrangement. A representative experiment is shown. Scale bar, 50 μm. Numbers indicate the mean ± SD of claudin-5 quantification across three replicate experiments. (**c**) Surface proteins in HUVECs were biotinylated followed by pull-down and immunoblotting for VE-cadherin. Surface VE-cadherin was reduced following treatment for 24 h with *db/db* vs *db/+* mouse sEVs (isolated from an equal volume of plasma). VEGF was included as a positive control for VE-cadherin internalisation. A representative experiment is shown. (**d**) Transcytosis measurements using TIRF microscopy of FITC-labelled albumin movement in lung microvascular ECs. sEVs from diabetic mice and humans (isolated from an equal volume of plasma) enhanced the number of transcytosis events (data from *n*=3 mouse sEV samples and *n*=4 human sEV samples with 12 transcytosis measures per treatment group are shown). ANOVA analysis was performed; *p<0.05, **p<0.01

Considering that EC barriers are upheld by various intercellular junction complexes, such as adherens and tight junctions, which govern paracellular leak, increased permeability could arise from alterations in cell health (e.g. cell death) or structural modulation. To address the former, TUNEL staining was employed and demonstrated that the increase in permeability did not appear to be due to enhanced cell death, as there were very few apoptotic cells in HUVECs treated with sEVs from *db/*+ or *db/db* mice (ESM Fig. 6a). To investigate whether sEVs in the diabetic state disrupt adherens or tight junctions, we performed immunofluorescence staining of vascular endothelial cadherin (VE-cadherin) or claudin-5, respectively, in HUVECs treated for 24 h with sEVs isolated from diabetic or non-diabetic individuals (Fig. 6a). Notably, VE-cadherin staining at cell–cell junctions appeared to be thinner in cells exposed to sEVs from diabetic individuals and there were regions where the barrier appeared to be disrupted (denoted by arrowheads in Fig. 6a). Staining for the tight junction protein, claudin-5, was also reduced in HUVECs treated with sEVs from diabetic individuals (Fig. 6b). A similar effect on VE-cadherin staining was apparent in HUVECs treated with *db/db* mouse sEVs compared with *db/*+ mouse sEVs (ESM Fig. 6b). Surface biotinylation experiments revealed that treatment with *db/db* mouse sEVs led to a reduction in surface VE-cadherin when compared with treatment with *db/*+ mouse sEVs (Fig. 6c).

Since phospho-proteomic analysis of treated ECs suggested that the actin cytoskeleton may also be impacted by sEVs in diabetes, we visualised the actin cytoskeleton with phalloidin staining. Importantly, junctional complexes interact with the actin cytoskeleton to regulate barrier formation [34]. Cortical actin staining appeared to be reduced in HUVECs treated with sEVs from diabetic individuals in areas near the cell membrane where VE-cadherin staining was punctate (indicated by arrowheads in Fig. 6a). To further test the impact of diabetic sEVs on actin dynamics, live imaging experiments were performed. Treatment of HUVECs with sEVs from diabetic individuals reduced cell protrusions compared with sEVs from non-diabetic individuals (ESM Fig. 7). Therefore, in diabetes, sEVs appear to impair actin dynamics, which may impact paracellular leak.

In addition to paracellular pathways, transport of material across the cell through the process of transcytosis can also affect barrier function [36, 37]. We performed total internal reflection fluorescence (TIRF) microscopy in primary human lung microvascular ECs to assess the impact of sEVs on transcytosis. Notably, sEVs isolated from diabetic mice or humans significantly enhanced transcytosis events (Fig. 6d).

### Plasma sEVs isolated from mice and humans in the diabetic state induce leak through MAPK/ERK and ROCK signalling

**Fig. 7:**
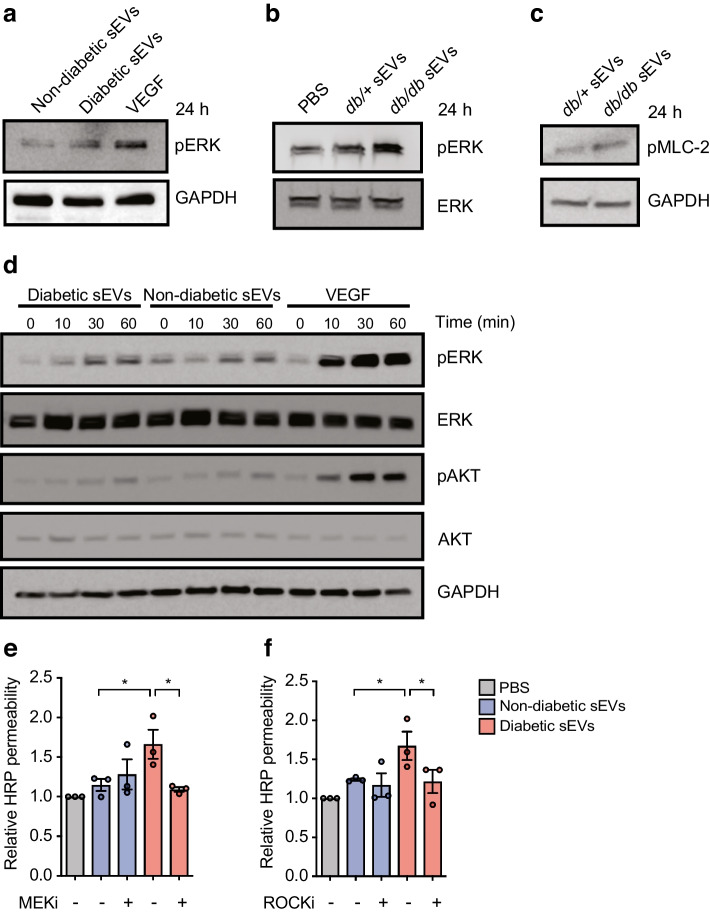
sEVs induce MEK and ROCK signalling, and inhibition of these pathways prevents leakage. (**a**) Immunoblotting for pERK with GADPH included as a loading control in HUVECs treated with sEVs isolated from an equal volume of plasma from non-diabetic or diabetic individuals. VEGF was included as a positive control for pERK activation. (**b**) Immunoblotting for pERK and total ERK in HUVECs treated with PBS^−/−^ or with sEVs from *db/*+ or *db/db* mouse plasma. (**c**) Immunoblotting for phosphorylated MLC-2 (pMLC-2) with GAPDH as a loading control in HUVECs treated with sEVs from an equal volume *db/*+ or *db/db* mouse plasma. (**d**) Time course analysis of ERK and AKT activation by immunoblot in HUVECs treated with sEVs from an equal volume of plasma from diabetic or non-diabetic individuals, or with VEGF as a positive control. pERK levels were increased after 30 min of treatment with plasma sEVs from diabetic individuals, with the increase being greater than that resulting from treatment with plasma sEVs from non-diabetic individuals. (**e**, **f**) HUVECs were pre-treated with a MEK inhibitor (SL327) (**e**) or a ROCK inhibitor (Y-27632) (**f**) and permeability was assessed in response to treatment with sEVs from non-diabetic or diabetic individuals (SEC-isolated from an equal volume of plasma, 1 h, HRP). While MEK or ROCK inhibition did not affect leakage in cells exposed to sEVs from non-diabetic individuals, these inhibitors blocked the increased permeability in response to sEVs from diabetic individuals (*n*=3 biological replicates). Data in (**e**, **f**) are presented as the mean ± SD. ANOVA analysis was performed; *p<0.05. Representative experiments are shown for all immunoblots. MEKi, MEK inhibitor; ROCKi, ROCK inhibitor

Pathway analysis predicted that the MAPK and Rho-GTPase pathways would be activated by sEVs in diabetes (Fig. 5f). To formally test this, phosphorylation of ERK (pERK) was assessed in HUVECs treated with sEVs from the plasma of diabetic or non-diabetic individuals (Fig. 7a) or *db/db* or *db/*+ mice (Fig. 7b) for 24 h. Both pERK levels (Fig. 7a,b) and phosphorylation of myosin light chain (MLC)-2 (Fig. 7c), a downstream target of ROCK, were increased in response to plasma sEVs isolated from the diabetic state. Assessing the kinetics of MAPK activation revealed that pERK levels were induced within 30 min in HUVECs exposed to sEVs from diabetic individuals (Fig. 7d). In contrast, phosphorylation of AKT was only modestly induced, and no differences were noted between sEVs from diabetic vs non-diabetic individuals. Finally, to determine whether the MAPK and p-MLC signalling pathways were functionally involved in leakage, HUVECs were exposed to MAPK kinase (MEK) or ROCK inhibitors prior to treatment with sEVs from non-diabetic or diabetic individuals. Inhibition of either of these pathways inhibited leakage induced by sEVs from diabetic individuals but did not alter leak in cells exposed to sEVs from non-diabetic individuals (Fig. 7e,f).

## Discussion

We demonstrate that plasma sEVs play a direct role in type 2 diabetes-induced vascular barrier disruption. Our observations highlight the considerable scope by which changes in systemic sEV characteristics can influence vascular complications in metabolic pathologies. While the origin of sEVs in the setting of diabetes has not been explored, studies have been done on their larger counterparts (i.e. microparticles). In particular, increases in the concentration of circulating proinflammatory microparticles, predominantly of platelet, leucocyte or EC origin, have been observed in diabetes [38, 39]. Mixtures of small and large EVs from diabetic individuals can induce pericyte detachment and blood–retina barrier disruption but the mechanisms have not been pursued [40]. Additionally, circulating microparticles induce cell death through activation of the complement cascade and a decrease in endothelial nitric oxide synthase, thereby increasing permeability [39, 41]. Notably, we did not observe EC death or inflammatory signalling, suggesting a distinct mechanism of action for sEVs isolated from the circulation of diabetic individuals.

In addition to direct cell-surface interactions, EVs can exert their functional effects by being internalised and releasing their cargo, including proteins, mRNAs and non-coding RNAs [42]. Analysis of the protein contents of sEVs suggested that the rapid disruption of EC permeability may be mediated through protein-mediated signalling, albeit specific EV-associated proteins responsible for inducing increased permeability in ECs were not identified. The maintenance of EC barrier integrity is a highly dynamic process involving the convergence of multiple pathways that prevent paracellular and transcellular extravasation of plasma and its macromolecular contents [33, 43]. In response to leak-inducing environmental cues, junctional components rapidly dissociate, internalise and are degraded, driving permeability [33, 43]. Increased tension on the actin cytoskeleton (e.g. through Rho-GTPases) can also result in actinomyosin contraction, actin stress fibre formation and cellular retraction, all of which destabilise junctions [43]. ROCK inhibitors have been shown to inhibit ras homolog family member A (RhoA) activity and MLC phosphorylation and, subsequently, reduce vascular permeability [44]. MEK signalling has also been implicated in mediating permeability [45], regulated in part by MLC kinase activity [46]. Our mechanistic studies revealed that sEVs in the diabetic state signal through the MEK and ROCK pathways to induce leakage, accompanied by altered cell–cell junctions and actin rearrangements. Future diabetes therapies could therefore target specific pathways activated by sEVs, including MEK and ROCK. Encouragingly, fasudil, a potent ROCK inhibitor, improves microvascular dysfunction in animal models of diabetes [47] and has shown promise in humans with diabetic macular oedema [48].

It is also increasingly appreciated that macromolecules can pass through ECs via transcytosis [43]. Transcytosis is particularly important in the BBB, where transcellular permeability is low [36]. Importantly, brain injury [49] and the ageing process itself [50] are associated with a compromised vascular barrier in the central nervous system due to enhanced transcytosis. Intriguingly, our study revealed that circulating sEVs from diabetic individuals enhanced transcytosis events in ECs. The relative contribution made by alterations to paracellular or transcellular permeability pathways remains to be determined. Taken together, our studies suggest an important contribution from sEVs in mediating vascular barrier disruption. Future studies should assess the generalisability of our findings to female mice and to other models of diabetes and explore whether barrier stabilising in the setting of diabetes may address the cognitive decline prevalent in the diabetic population.

### Supplementary Information

Below is the link to the electronic supplementary material.

Supplementary file1 (PDF 6228 KB)

## Data Availability

The authors confirm that the data supporting the findings of this study are available within the article and its supplementary materials. Further information and requests for resources or reagents may be sent to the corresponding author.

## Abbreviations

ALIX: ALG-2-interacting protein X
BBB: Blood–brain barrier
Cryo-EM: Cryogenic electron microscopy
EC: Endothelial cell
EV: Extracellular vesicle
HRP: Horseradish peroxidase
MEK: MAPK kinase
MLC: Myosin light chain
NTA: Nanoparticle tracking analysis
PBS^−/−^: Dulbecco’s PBS without Ca^2+^ or Mg^2+^
RAS: Rat sarcoma
ROCK: Rho-associated protein kinase
SEC: Size-exclusion chromatography
sEV: Small extracellular vesicle
TIRF: Total internal reflection fluorescence
VE-cadherin: Vascular endothelial cadherin
VEGF: Vascular endothelial growth factor
