# Supplementary material for: Circulating small extracellular vesicles mediate vascular hyperpermeability in diabetes
Source: Diabetologia. 2024 Mar 15;67(6):1138–54. doi: 10.1007/s00125-024-06120-9 (PMC11058313; doi:10.1007/s00125-024-06120-9)
Supplement: Supplementary file 1 — Supplementary file1 (PDF 6228 KB) [file 125_2024_6120_MOESM1_ESM.pdf]

Electronic Supplementary Material (ESM)

TITLE: Circulating small extracellular vesicles mediate vascular hyperpermeability in diabetes

SHORT TITLE: Diabetic hyperpermeability induced by extracellular vesicles

AUTHORS: Dakota Gustafson[1,2*], Peter V. DiStefano[1*], Xue Fan Wang[3], Ruilin Wu[1,2], Siavash Ghaffari[4], Crizza Ching[1,5], Kumaragurubaran Rathnakumar[1], Faisal Alibhai[1], Michal Syonov[4], Jessica Fitzpatrick[6], Emilie Boudreau[1], Cori Lau[1,2], Natalie Galant[1], Mansoor Husain[1,2], Ren-Ke Li[1], Warren L. Lee[2,4,7], Rulan S. Parekh[6], Philippe Monnier[3,8,9], and Jason E. Fish[1,2,5,10]

[*]Dakota Gustafson and Peter V. DiStefano contributed equally to this manuscript.

AFFILIATIONS: [1]Toronto General Hospital Research Institute, University Health Network, Toronto, Canada, [2]University of Toronto, Department of Laboratory Medicine and Pathobiology, Toronto, Canada, [3]Division of Fundamental Neurobiology, Toronto Western Research Institute, University Health Network, Toronto, Canada, [4]Keenan Research Centre for Biomedical Science, St. Michael's Hospital, Toronto, Canada, [5]University of Toronto, Institute of Medical Science, Toronto, Canada, [6]Department of Medicine and Pediatrics, Women's College Hospital, Hospital for Sick Children and University of Toronto, [7]Department of Biochemistry, University of Toronto, Toronto, Canada; [8]Donald K. Johnson Eye Institute, Krembil Research Institute, University Health Network, Toronto, Canada, [9]Department of Physiology, Faculty of Medicine, University of Toronto, Canada, [10]Peter Munk Cardiac Centre, University Health Network, Toronto, Canada

ADDRESS CORRESPONDENCE TO:

Dr. Jason Fish, Ph.D.
Toronto General Hospital Research Institute
University Health Network
101 College Street, 3-309 Princess Margaret Cancer Research Tower
Toronto, Ontario, Canada, M5G 1L7
Email: Jason.Fish@utoronto.ca
Tel: +1(416)-522-8191
Fax: +1(416)-581-7484

SUPPLEMENTAL NONSTANDARD ABBREVIATIONS AND ACRONYMS:

**ERK**	Extracellular signal-regulated kinase
**ESM**	Electronic supplementary material
**FBS**	Fetal bovine serum
**FDR**	False-discovery rate
**FITC**	Fluorescein isothiocyanate
**HRP**	Horseradish peroxidase
**hCMEC/D3**	Human cerebral microvascular endothelial cells
**HUVEC**	Human umbilical vein endothelial cells
**K_2EDTA**	Dipotassium ethylenediaminetetraacetic acid
**kDa**	Kilodalton
**NF-κB**	Nuclear factor κ-light-chain-enhancer of activated B cells
**NTA**	Nanoparticle tracking analysis
**PBS**	Phosphate buffered saline
**RT**	Room temperature
**SEC**	Size-exclusion chromatography
**sEVs**	Small extracellular vesicles
**TBS**	Tris-buffered saline
**TIRF**	Total internal reflection fluorescence
**VE-cadherin**	Vascular endothelial cadherin
**VEGF**	Vascular endothelial growth factor

ESM METHODS:

Human study participants:

The study implemented a sample matching procedure based on age and biological sex. Age matching was executed within a one-year range, ensuring precision in participant alignment, while identical sex-matching (self-reported) also occurred. Samples were initially collected from participants enrolled at a single site from the Family Investigation of Nephropathy and Diabetes study (NCT00301249) who had no evidence of advanced kidney disease from the African American community.

Murine models:

In vivo multiphoton microscopic intravital imaging and leakage quantification: Anaesthetisation of mice was facilitated by intra-peritoneal injection of tribromoethanol avertin (Sigma-Aldrich, Oakville, ON, Canada) at a dose of 250 $g.kg^{-1}$. Toe pinch was used to assess the level of sedation prior to surgery. Post-sedation, 100 µl (2.5 mg/ml^{-1}) of bupivacaine (Thermo Fisher Scientific, Waltham, MA, US) was injected into the scalp for supplemental local anaesthesia and the scalp skin was then removed. Mice were mounted onto a stereotaxic frame (Narishige, Tokyo, Japan) with ear bars (EB-3B, Narishige, Tokyo, Japan), stabilising the head to prevent any movement. A craniotomy was then performed with a high-speed micro-drill (Harvard Apparatus, Holliston, MA, USA) to create a 1 cm (diameter) circular optical window over the parietal cortex. Cranial sutures were carefully avoided to prevent overt damage to the brain. After the removal of the scalp, the surface of the cortex was rinsed with sterile PBS. A Vaseline wall was then applied around the cranial opening and 0.75% (w/v) agarose (Sigma-Aldrich, Oakville, ON, Canada) was added into the Vaseline wall. An 8 mm diameter round-cover slip (VWR, Radnor, PA, USA) was immediately added to the top of the agarose to form an imaging window. Mice were injected with 150 µl of a mixture of Texas Red-70 kDa dextran (Thermo Fisher Scientific, Waltham, MA, USA) and FITC-10 kDa dextran (Thermo Fisher Scientific, Waltham, MA, USA) at a concentration of 10 mg/ml^{-1} via retro-orbital injection. For small extracellular vesicle (sEV) injections, after a baseline was established for the tracers, mice were subsequently injected with 150 µl of sEVs isolated from *db/+* or *db/db* mice by retroorbital injection. The experimenter was blinded to the grouping of each sEV sample. Cerebral vessels were imaged using a Leica (Leica, Wetzlar, DE) two-photon microscope equipped with 25x water-immersion objective lenses. FITC and Texas Red fluorophore was excited at 900 nm wavelength with approximately 5% laser power and emission was detected with 500-600 nm and 700-800 nm optical filters respectively. Imaging fields were randomly selected with z-stack of approximately 200 µm deep into the cortical surface; scanning speed was set to 700. Digital gain was kept constant and laser power was micro-adjusted to avoid pixel saturation. Time-lapse images were continuously taken for a total duration of 45 minutes. Acquired z-stack images were maximum intensity projected using Fiji-ImageJ. Rigid body image registration was done using StackReg plugin on Fiji-ImageJ. Vessel segmentation was quantified using k-mean clustering. Segmentation output was assessed manually, and extravascular space was selected as region-of-interest upon threshold-based filtering. Total extravascular leakage intensity of the 45 minutes time frame (t45) was quantified and normalised to total thresholding area.

Enrichment, analysis and quality control of circulating sEVs from blood plasma:

Preparation of murine and human plasma samples: Human – Peripheral blood samples were collected synchronously with the standard-of-care cardiac bloodwork, with the first tubes being utilised for essential clinical bloodwork [1]. Peripheral blood samples for research (10 ml) were drawn from the cubital vein into BD Vacutainer® Blood Collection Tubes (BD Biosciences, San Jose, CA, USA) containing dipotassium ethylenediaminetetraacetic acid (K_2EDTA) using a 21-gauge needle and processed within three hours. Plasma was separated from whole blood through centrifugation (2,000×g, room temperature [RT], 15 minutes) and stored at -80°C until downstream processing. At no time during the process was the plasma subjected to temperatures below 4°C or above 25°C. Samples were thawed on ice and subjected to sequential centrifugation of (2,500×g, 4°C, 15 minutes) to assist in the reduction of platelet counts and large particulates according to the recommended International Society on Thrombosis and Haemostasis protocol [2]. Samples underwent a single freeze-thaw cycle prior to experimental utilisation. Murine – Isolation of murine blood and derivatives was carried out in the morning using a dedicated murine operating room. Briefly, 14-week-old mice were anesthetised through vaporized isoflurane (Dechra, Overland Park, KS, USA) and placed on a rodent warming pad (Thermo Fisher Scientific, Hampton, NH, USA) to maintain body temperature. Induction was carried out using 3-4% isoflurane to ensure maintenance of "deep" anesthetic plane which was assessed using the pedal reflex as per institutional protocols. Following this, 23-gauge BD Precision Glide® syringe needles (BD Biosciences, San Jose, CA, USA) containing aspirated 0.5 mol/l K_2EDTA (EMD Millipore, Burlington, MA, USA) were used to facilitate collection of blood via cardiac puncture of the right ventricle; exsanguination as a result of the terminal cardiac puncture occurred on average four minutes after confirmation of anesthetic plane. Processing – Plasma was separated from whole blood through centrifugation (2,000×g, RT, 20 minutes), with the platelet-rich plasma being carefully separated from other blood components. Prior to freezing, platelet-rich plasma samples were sequentially centrifuged (2,500×g, 4°C, 15 minutes; 5810r Centrifuge, Eppendorf, Framingham, MA, United States) to facilitate platelet reduction (i.e., generate platelet-poor plasma).

All samples were stored at -80°C in protein low bind tubes (Eppendorf, Framingham, MA, United States) until downstream utilisation. At no time during the process was the blood, or derivatives of such, subjected to temperatures below 4°C or above 25°C. Hemolysis was examined prior to downstream analysis by measuring the absorbance at 414 nm using a DS-11$^+$ Spectrophotometer (DeNovix, Wilmington, DE, United States), with samples meeting exclusion criteria if they were greater than one standard deviation from the mean; no samples were excluded. Details of plasma and sEV collection, according to the MIBlood-EV standardised reporting tool [3] are indicated below.

Nanoparticle tracking analysis: sEV samples were diluted in ice-cold 0.22 µm filtered Dulbecco's phosphate buffered saline (PBS)$^{-/-}$ and analysed through nanoparticle tracking analysis (NTA) using a NanoSight NS300 system (Malvern Panalytical Ltd, Amesbury, UK) configured with a 488 nm laser module and a high sensitivity scientific complementary metal-oxide-semiconductor camera (OrcaFlash2.8, Hamamatsu C11440, NanoSight Ltd). Prior to analysis, sEV aggregates were broken up by gentle passage through a 29-gauge needle and were treated with either 0.1% (v/v) Triton X-100 (Sigma-Aldrich, Oakville, ON, Canada) [4] for 10 minutes at room temperature

(RT), or mock-treated with a matching volume of 0.22 µm filtered Dulbecco's PBS$^{-/-}$ (control). Analysis using NTA software (version 3.2, build 16) closely followed experiments previously described [5]. In brief, diluted sEVs were introduced at a rate of 75 (~7.8 µl/minute) using a syringe pump (Harvard Apparatus, Holliston, MA, USA), yielding an x-drift of ~-3.0 pixels/frame. The temperature for the measurements was kept consistently at 25°C with a viscosity of 0.9 cP (water). Sample dilutions were conducted such that an average of 34 particles/frame were in the field of view at all times (linear dynamic range of 20-100 particles/frame). After optimisation, settings were kept constant between measurements for the study (ESM Table 2). Three one-minute duration videos, with a 30-second initialisation delay, were recorded for each independent biological replicate, generating data for each sample type that is the average of 42-45 individual measurements. Polystyrene beads of 100 nm and 200 nm (Malvern Panalytical Ltd, Amesbury, UK) in diameter were used as a calibrator.

Transmission electron cryomicroscopy: Cryogenic electron microscopy samples were prepared using Quantifoil R 2/2 copper grids (Electron Microscopy Sciences, Hatfield, PA, USA) and negatively glow discharged in ambient air. Concentrated sEV sample preparations (5 µl) were directly applied to grids and incubated in a Vitrobot (Field Electron and Ion Company, Eindhoven, NL) at 100% relative humidity (blot time 1.5-2.0 seconds, 4°C) before being plunged frozen into liquid ethane. Vitrified samples were imaged using a Talos L120C transmission electron microscope (Thermo Fisher Scientific, Waltham, MA, USA) with a 4,096×4,096-pixel complementary metal-oxide-semiconductor camera at a temperature between -173°C and -175°C.

INTERNATIONAL SOCIETY for EXTRACELLULAR VESICLES

MIBlood-EV

Standardized Reporting Tool for Blood EV Research (Human)

STUDY INFORMATION

1.0 **Manuscript title**	Circulating Small Extracellular Vesicles Mediate Diabetic Vascular Hyperpermeability		
1.1 **Corresponding author (Name and Email)**	Jason Fish (Jason.Fish@utoronto.ca)		
1.2 **Institution name**	Toronto General Hospital Research Institute / University Health Network		
1.3 **Time period of experiment (years)**	2018-2023	1.4 **Number of samples**	30
1.5 **Cargo of interest**	✔ Vesicles ✔ Protein ☐ RNA ☐ DNA ☐ Other:		
1.6 **Biospecimen type**	✔ Plasma ☐ Serum	1.7 **Biospecimen state**	Frozen
1.8 **Source of frozen specimens**	Obtained from collaborat[illegible]	1.9 **Years of collection (range)**	

BLOOD COLLECTION AND PROCESSING

2.0 **Patient fasting status**	Uncertain	2.1 **Fasting length (e.g. hours/days)**	N/A
2.2 **Anatomical access site**	Cubital vein	2.3 **Needle diameter (e.g. gauge)**	21
2.4 **Blood volume collected (mL)**	10		
2.5 **Plasma anticoagulant**	✔ EDTA ☐ Citrate ☐ Heparin ☐ Other:		
2.6 **Serum tube type**		2.7 **Serum clotting time (minutes)**	
2.8 **Time between collection and first centrifugation (range in hours)**	Unknown		
2.9 **Transport temperature**	Room temp (~2[illegible]	2.10 **Transport condition of tubes**	Vertical
2.11 **Centrifuge brand and model**	5810r Centrifuge, Eppendorf, Framingham, MA, United States		
2.12 **Bucket rotor type**	Swing	2.13 **Number of centrifugation cycles**	Two
2.14 **1st Centrifugation speed (RCF in x g)**	2,500	2.15 **1st Rotor brake**	Slow
2.16 **1st Centrifugation temperature**	4°C	2.17 **2nd Centrifugation speed (RCF in x g)**	2,500
2.18 **2nd Rotor brake**	Slow	2.19 **2nd Centrifugation temperature**	4°C
2.20 **Additional processing steps (e.g. filtration)**	N/A		
2.21 **Storage tubes (brand, type, source, catalog number)**	Eppendorf, 1.5mL Low Bind, MedStore, 22431021		
2.22 **Storage temperature**	-80°C	2.23 **Length of storage (range in years)**	0-2

PLASMA/SERUM QUALITY CONTROL

3.0 **Number of freeze-thaw cycles (range)**	1		
3.1 **Thawing temperature**	on ice	3.2 **Thawing duration (minutes)**	60

Hemolysis

3.3 **Presence of hemolysis**	No	3.4 **Number of samples affected (e.g. <25%, 25-50%)**	<1%
3.5 **Method used**	Spectrophotometry	3.6 **RBC count (Median, 95% CI, N)**	
3.7 **RBC counter brand and type**			
3.8 **Spectrophotometry hemoglobin concentration (g/L)**			
3.9 **Spectrophotometer brand, model and wavelength measured (e.g. 414 nm)**	DeNovix, DS-11+ Spectrophotometer, 414nm		
3.10 **Hemolized samples were discarded**	No		

Platelets

3.11 Presence of platelets	Not Tested	3.12 Method used (e.g. Flow Cytometry)	
3.13 Marker(s) used (e.g. CD61, CD41)			
3.14 Concentration (median, 95% CI, N)			
3.15 Platelet counter instrument brand and type			
3.16 Flow cytometer brand and type			
3.17 Flow cytometry size and fluorescence ranges of detection in nanometers and MESF, respectively			

Lipoproteins

3.18 Presence of lipoproteins	Yes	3.19 Method used (WB, ELISA, FC)	Other: Beckman AU48[illegible]
3.20 Spectrophotometry L-index			
3.21 Spectrophotometer brand, model and wavelength measured (e.g. 700 nm)			
3.22 WB Marker(s) used (e.g. Apo B)			
3.23 Western blot images provided in manuscript?			
3.24 Flow cytometry marker(s) used (e.g. ApoB)			
3.25 Flow cytometry concentration (median, 95% CI, N)			
3.26 Flow cytometer brand and type			
3.27 Flow cytometry size and fluorescence ranges of detection in nanometers and MESF, respectively			

ESM METHODS – Continued:

Biochemistry/gene expression experiments:

Clinical chemistry: A Beckman AU480 Biochemistry Analyzer (Beckman Coulter, Pasadena, CA, USA) was utilised to colorimetrically/photometrically determine the levels of total cholesterol, triglycerides, low-density lipoprotein cholesterol, high-density lipoprotein cholesterol levels, and glucose.

sEVmass spectrometry: A total of 40 µl of sEVs were loaded onto a single-stacking gel band to remove lipids, detergents, and salts. The gel band was reduced with dithiothreitol (Sigma-Aldrich, Oakville, ON, Canada), alkylated with iodoacetic acid, and digested with trypsin (Promega, San Luis Obispo, CA, USA). Extracted peptides were resolubilised in 0.1% (v/v) aqueous formic acid (Thermo Fisher Scientific, Waltham, MA, USA) and loaded onto a Thermo Acclaim Pepmap (75 µm ID X 2 cm C18 with 3 µm beads) pre-column (Thermo Fisher Scientific, Waltham, MA, USA) and then onto an Acclaim Pepmap Easy spray (75 µm X 15 cm with 2 µm C18 beads) analytical column (Thermo Fisher Scientific, Waltham, MA, USA) and separated using a Dionex Ultimate 3000 uHPLC (Thermo Fisher Scientific, Waltham, MA, USA) at 220 nl/ml^{-1} with a gradient of 2-35% organic 0.1% (v/v) formic acid in acetonitrile over two hours. Peptides were analysed using a Thermo Orbitrap Fusion mass spectrometer (Thermo Fisher Scientific, Waltham, MA, USA) operating at 120,000 resolution for MS1 with HCD sequencing at top speed (15,000 resolution) for all peptides with a charge of 2^{+} or greater.

Phosphoproteomics: Approximately 750,000 HUVEC were plated on 60 mm tissue culture plates (Corning, Costar, New York, NY, USA) coated with 10 µg/ml^{-1} fibronectin and grown to confluency, followed by treatment with 160 µl sEVs from non-diabetic or diabetic human plasma for one hour. Cell were washed with ice-cold TBS and lysed in 1X RIPA buffer (EMD Millipore, Burlington MA, USA) and spun at 17,000×g for five minutes. Proteins were precipitated out of the lysis buffer using -80°C acetone (Sigma-Aldrich, Oakville, ON, Canada) precipitation for one hour. The protein pellet was incubated with 8 mol/l urea (Sigma-Aldrich, Oakville, ON, Canada) and 100 mmol/l ammonium bicarbonate (Sigma-Aldrich, Oakville, ON, Canada), Protease Max (Promega, San Luis Obispo, CA, USA), and dithiothreitol (Sigma-Aldrich, Oakville, ON, Canada) for one hour at 55°C to reduce the samples. The digested proteins were alkylated with acrylamide (Sigma-Aldrich, Oakville, ON, Canada) and digested overnight with Trypsin/Lys-C (Promega, San Luis Obispo, CA, USA) at 37°C. After digestion, the reaction was quenched using 50% (v/v) formic acid (Sigma-Aldrich, Oakville, ON, Canada). The digested pellets were spun down at 10,000×g for two minutes and the pellet was dried using a speed vac. The pellet was enriched for phospho-peptides using the High-Select Fe-NTA Phosphopeptide Enrichment Kit (Thermo Fisher Scientific, Waltham, MA, USA) per manufacturer's instructions. Raw mass spectrometry data were converted into *.mgf format (Mascot generic format) for searching using the Mascot 2.5.1 search engine (Matrix Science) against human and mouse protein sequences (Uniprot 2019). The database search results were loaded onto Scaffold (v_5.3; Proteome Software Inc) for statistical assessment and data visualisation.

Proteomics and pathway analysis: Pathway analysis was conducted using the Max Plank Institute for Molecular Genomics Consensus PathPB-human/mouse and tested for enrichment by a

hypergeometric test with adjustment for multiple comparisons using the Benjamini-Hochberg False Discovery Rate (FDR), with Q<0.05 considered to be statistically enriched in a gene set of interest [6-8]. Although many hypotheses were tested throughout the manuscript, no experiment-wide multiple-testing correction was applied. Raw mass spectrometry data were converted into *.mgf format (Mascot generic format) for searching using the Mascot 2.5.1 search engine (Matrix Science) against human and mouse protein sequences (Uniprot 2019). The database search results were loaded onto Scaffold (v_5.3; Proteome Software Inc) for statistical assessment and data visualisation.

RNA isolation and qRT-PCR: RNA isolation was performed using a RNeasy Mini Kit (Qiagen, Germantown, MD, USA) according to the manufacturer's instructions. Complementary DNA was obtained with multiscribe reverse transcriptase (Applied Biosystems, Foster City, CA, USA) and Oligo(dT) primers (Invitrogen, Carlsbad, CA, USA) from 1 µg of input RNA and Oligo dT primers. Amplifications were run on a Roche 480 Instrument II Lightcycler (Roche Diagnostics, Mississauga, ON, Canada) using previously published murine and human primer sets (sequences can be found in ESM Table 3). Each value was calculated using the ΔΔCq method and normalised to *GAPDH* as an internal control.

NF-κB reporter assay: Cells were grown to 70-80% confluence on 10 µg/ml^{-1} fibronectin coated 24-well tissue culture plates (Costar, Corning, NY, USA) and transfected, as described previously [9]. Briefly, hCMEC/D3 cells were transfected with 2 µg of NF-κB reporter luciferase construct (containing 5x NF-κB elements; Promega, San Luis Obispo, CA, USA) and 50 ng of pRL Renilla luciferase construct (Promega, San Luis Obispo, CA, USA), using Lipofectamine 2000 (Thermo Fisher Scientific, Waltham, MA, USA). Cell lysates were isolated after treatments using passive lysis buffer (Promega, San Luis Obispo, CA, USA) and luciferase activity was assessed using the Dual Luciferase Reporter Assay System (Promega, San Luis Obispo, CA, USA) using a GloMax Luminometer (Promega, San Luis Obispo, CA, USA).

Western blotting: Concentrated sEV samples or cell lysates were mixed with radioimmunoprecipitation assay buffer ([RIPA], EMD Millipore, Burlington MA, USA) containing Complete™ protease inhibitor cocktail (Roche Life Science, Mississauga, ON, Canada) at the concentration recommended by the manufacturer and sonicated for five minutes, three times, with vortexing in between. Non-reducing Laemmli loading buffer (Bio-Rad, Philadelphia, PA, USA) was added to the lysed samples and volumes corresponding to 20 µg of protein from isolates were separated on a 4-20% mini-PROTEAN® TGX™ gel (Bio-Rad, Philadelphia, PA, USA) for sodium dodecyl sulphate polyacrylamide gel electrophoresis according to the manufacturer's standard protocol. Proteins were transferred to an Amersham™ Protran™ 0.45 µm Hybond polyvinylidene fluoride membrane (GE Healthcare, Chicago, IL, USA) by wet blotting. Next, non-specific binding sites were blocked using 5% (w/v) Blotting Grade Blocker Non-Fat Dry Milk (Bio-Rad, Philadelphia, PA, USA) in Tris-buffered saline ([TBS], Thermo Fisher Scientific, Hampton, NH, USA) for two hours. The membrane was then incubated with the following antibodies: mouse anti-human mAb ALIX (Santa Cruz Biotechnology, Dallas, TX, United States; 1:1000 dilution), mouse anti-human mAb CD63 (Santa Cruz Biotechnology, Dallas, TX, United States; 1:1000 dilution), rabbit anti-human pAb extracellular signal-regulated kinase (ERK)1/2 (p44/p42, Cell Signaling Technology, Danvers, MA, USA; 1:1000 dilution), rabbit anti-human mAb phospho-ERK1/2 (Thr202/Tyr204, Cell

Signaling Technology, Danvers, MA, USA; 1:2000 dilution), rabbit anti-human pAb AKT (Cell Signaling Technology, Danvers, MA, USA; 1:1000 dilution), rabbit anti-human mAb phospho-AKT (Ser473, Cell Signaling Technology, Danvers, MA, USA; 1:1000 dilution), mouse anti-human mAb GAPDH (Santa Cruz Biotechnology, Dallas, TX, United States; 1:4000 dilution), rabbit anti-human mAb Vascular Endothelial (VE)-Cadherin (Cell Signaling Technology, Danvers, MA, USA; 1:1000 dilution), or rabbit anti-human pAB phospho-myosin light chain (Thr18/Ser19, Cell Signaling Technology, Danvers, MA, USA; 1:1000 dilution) diluted in 5% Blotting Grade Blocker Non-Fat Dry Milk (BioShop, Burlington, ON, CA) in TBS-Tween (TBST, Sigma-Aldrich, Oakville, ON, Canada) overnight at 4°C. Following incubation, the membrane was washed with TBST for 10 minutes, three times. Detection was then facilitated by incubating the membrane with the following species matched horseradish peroxidase (HRP) crosslinked antibodies: goat anti-rabbit IgG, HRP-linked (Cell Signaling Technology, Danvers, MA, USA; 1:3000 dilution) or horse anti-mouse IgG, HRP-linked (Cell Signaling Technology, Danvers, MA, USA; 1:3000 dilution) diluted in 5% Blotting Grade Blocker Non-Fat Dry Milk (BioShop, Burlington, ON, CA) in TBST for one hour at RT. All antibodies have been previously validated and bands were of the expected size and were not present in the absence of primary antibody. Chemiluminescent detection was performed following a five-minute incubation with SuperSignal West Pico PLUS™ Chemiluminescent Substrate (Thermo Fisher Scientific, Waltham, MA, USA). Signal was subsequently captured on a ChemiDoc (Bio-Rad, Philadelphia, PA, USA).

VE-cadherin internalisation assay: After treatments, cells were washed with ice-cold PBS and incubated with 0.5 mg/ml^{-1} NHS-Sulfo-Biotin (BioVision, Milpitas, CA, USA) for 45 minutes on ice with rocking at 4°C. The biotin solution was aspirated, and unbound biotin quenched by washing cells three times with ice-cold 50 nmol/l glycine (Thermo Fisher Scientific, Waltham, MA, USA). Cells were then washed with PBS, lysed in 1X RIPA buffer, scraped into DNA low bind tubes (Eppendorf, Framingham, MA, United States), and clarified at 17,000×g_{avg} for five minutes. Lysates were then incubated with streptavidin sepharose (Cell Signaling Technology, Danvers, MA, USA) for four hours at 4°C. The sepharose was washed three times with 1X RIPA buffer and biotinylated proteins were eluted with 2X Laemmli buffer (Bio-Rad, Philadelphia, PA, USA) and boiled at 95°C for 10 minutes.

Cell biology experiments:

Cell Culture: Human umbilical vein endothelial cells (HUVEC, ScienCell, Carlsbad, CA, USA or PromoCell, distributed by Cedarlane, Burlington, ON, Canada) and human cerebral microvascular endothelial cells (hCMEC/D3, Cellutions Biosystems Inc, Toronto, ON, CA) were cultured in endothelial cell medium (ECM), 5% (v/v) fetal bovine serum (FBS), 1% (v/v) endothelial cell growth supplement (ECGS), and 100 µg/ml^{-1} penicillin-streptomycin (ScienCell, Carlsbad, CA, USA) at 37°C in a 5% CO_2 humidified incubator. Cells were maintained on tissue culture plates (Corning, Costar, New York, NY, USA) coated with either 0.1% (v/v) gelatin attachment factor (Thermo Fisher Scientific, Waltham, MA, USA; for HUVECs) or 10 µg/ml^{-1} type I rat tail collagen (Sigma-Aldrich, Oakville, ON, Canada; for hCMEC/D3s) and passaged every 2-4 days at 70-80% confluence. For experimentation, HUVEC at passages 3-7 and hCMEC/D3 cells at passages 32-35 were used. Mouse brain endothelial cells, referred to as b.End3 (ATCC, Manassas, VA, USA) were cultured in Dulbecco's Modified Eagle's Medium with L-glutamine (Thermo Fisher Scientific, Waltham, MA, USA), 10% (v/v) FBS, and 100 µg/ml^{-1} penicillin/streptomycin

(ScienCell, Carlsbad, CA, USA) at 37°C in a 5% CO_2 humidified incubator. The bEND.3 cells were cultured on attachment factor coated tissue culture plates and used from passages 12-14. All cells were purchased directly from the companies and were verified as correct by phenotypic characterisation. Source cell vials were monitored and subsequently tested negative for the presence of mycoplasma contamination (Thermo Fisher Scientific, Waltham, MA, USA).

sEV treatments: For cell biology experiments, sEVs (isolated by ExoQuick or size-exclusion chromatography [SEC]) isolated from an equal volume of plasma for each condition were added at a final concentration of ~0.5-1.0 x 10^{10} particles/ml in complete media (containing FBS depleted of bovine serum EVs) for the specific amount of time before analysis. EV depletion was facilitated through a 24-hour ultracentrifugation of FBS at 120,000×*g*, as per published recommendation [10].

Transwell leak assay: Endothelial monolayer leak assays were performed similar to previously described [11]. Briefly, HUVEC or bEND.3 cells were seeded on 3 µm pore transwell inserts (Corning Life Sciences, Corning, NY, USA) coated with 10 µg/ml^{-1} human plasma fibronectin (Sigma-Aldrich, Oakville, ON, Canada). The cells were grown on the inserts for two days until reaching 95% confluence. Subsequently, the cells were incubated in the concordant media containing FBS depleted of bovine serum EVs. Depletion was facilitated through a 24-hour ultracentrifugation of FBS at 120,000×g_{avg}, as per published recommendation [10]. Cells were subsequently treated acutely (one hour) or overnight (16-24 hours) with sEVs. For sEVs isolated by ExoQuick or SEC, 25 µl or 25-45 µl of sEVs were added, respectively in a 500 µl volume of media. A one-hour treatment with 50 ng/ml^{-1} vascular endothelial growth factor (VEGF, Sigma-Aldrich, Oakville, ON, Canada) was used as a positive control. After treatments, horseradish peroxidase (HRP, Sigma-Aldrich, Oakville, ON, Canadawas added into the upper chamber at a concentration of 1.5 µg/ml^{-1} and tracer flux permitted for one hour. The experimenter was blinded to the grouping of each sEV sample. HRP accumulation in the bottom chamber was assessed using a 3,3’,5,5’-Tetramethylbenzidine (TMB) colorimetric assay (Cell Signaling Technology, Danvers, MA, USA). Specifically, 10 µl aliquots of media from the lower chamber were placed in a 96-well plate in triplicate, to which 100 µl of TMB was added. The reaction was allowed to occur for one minute alongside a standard curve of HRP to empirically determine tracer concentration in the lower chamber. Termination of the reaction was facilitated by adding 100 µl of 1 mol/l hydrochloric acid (Sigma-Aldrich, Oakville, ON, Canada) and the absorbance was then read at 450 nm using a Biotek Cytation 5 (Biotek, Winooski, VT, USA). For some acute stimulation experiments with sEVs, 40 kilodalton (kDa) fluorescein isothiocyanate (FITC)-dextran (Sigma-Aldrich, Oakville, ON, Canada) was used as a tracer. Prior to leak quantification, cells were changed to Hanks Buffered Salt Solution (Thermo Fisher Scientific, Waltham, MA, USA) and 1 mg/ml^{-1} FITC-dextran was added with either sEVs (as above) or VEGF (50 ng/ml) as a positive control in the top chamber and tracer flux allowed to occur for one hour. For inhibitor experiments, cells were pre-treated for two hours with either 30 µmol/l of the mitogen-activated protein kinase (MEK) inhibitor SL327 (Sigma-Aldrich, Oakville, ON, Canada), 10 µmol/l of the Rho-Associated Kinase (ROCK) Inhibitor Y-27632 (MedChem Express, Monmouth Junction, NJ, USA), or dimethylsulfoxide as a vehicle control. For time-course measurements, aliquots from the bottom chamber of the transwell were taken at 15, 30, 60, 90, and 120 minutes. Monolayer leak was quantified through measurement of the fluorescent signal via excitation at 485 nm and emission at 535 nm using the same Biotek Cytation 5. Removal of sEV surface proteins was conducted by incubating sEVs with 0.5 mg/ml^{-1} proteinase K (Thermo Fisher Scientific, Waltham, MA, USA)

at 37°C for two hours; inactivation was facilitated by incubating the mixture at 50°C for 10 minutes.

xCELLigence measurement of barrier kinetics: Barrier properties were assessed using the xCELLigence real-time cellular analysis (RTCA) dual purpose (DP) System from ACEA/Agilent (Santa Clara, CA, USA). Experiments were performed in the E-Plate 16 (ACEA, Cat. #680612). Wells were coated with 10 µg/ml of fibronectin followed by addition of complete endothelial cell medium (ScienCell, Carlsbad, CA, USA). A blank measurement was made followed by the addition of 40,000 HUVEC/well. After 20 minutes, the E-plate was locked into the RTCA DP analyzer and measurements were made every 15 minutes. Cells were grown for ~24 hours until impedance readings reached a steady state (i.e., confluence). Readings were then made every 30 seconds for ~1 hour, which served as the baseline. PBS or sEVs (resuspended in PBS) from an equal volume of *db/*+ or *db/db* mouse plasma were added directly to the wells without pausing the analyzer and measurements were made for ~10 additional hours. Data was normalised to the PBS control.

Immunofluorescence microscopy: Cells were grown to confluence on 10 µg/ml^{-1} fibronectin-coated 8-well chamber slides (Sigma-Aldrich, Oakville, ON, Canada). Following sEV treatment or exposure to VEGF (50 ng/ml, 30 mins) or tumor necrosis factor-α (10 ng/ml, 30 mins; used as positive controls), cells were washed with PBS and fixed with 4% (v/v) paraformaldehyde for 20 minutes (Sigma-Aldrich, Oakville, ON, Canada). Cells were washed again with PBS and permeabilised with 0.2% (v/v) Triton X-100 (Sigma-Aldrich, Oakville, ON, Canada) for five minutes and subsequently blocked with 5% (v/v) BSA/PBS-T for one hour. Permeabilised cells were then incubated with the primary antibodies overnight (16 hours) at 4°C and secondary antibody for two hours at RT away from light. The primary antibodies used were: mouse anti-human mAb VE-cadherin (R&D Systems, Minneapolis, MN, USA), rabbit anti-mouse pAb Claudin-5 (Thermo Fisher Scientific, Waltham, MA, USA), rabbit anti-human mAb Nuclear factor κ-light-chain-enhancer of activated B cells (NF-κB) p65 (Cell Signalling, Danvers, MA, United States). Secondary antibodies used were: rabbit anti-mouse IgG-AlexaFluor 488 (Thermo Fisher Scientific, Waltham, MA, USA) and goat anti-rabbit IgG-AlexaFluor 488 (Thermo Fisher Scientific, Waltham, MA, USA). To stain for the actin cytoskeleton, cells were incubated with 165 nM Rhodamine-phalloidin (Thermo Fisher Scientific, Waltham, MA, USA) for 30 minutes at RT away from light. The chamber slides were mounted, and the cells were counterstained with mounting media containing DAPI (Vector Labs, Burlingame, CA, USA) onto 24x50 mm No.1 Coverslips (VWR, Radnor, PA, USA). Images were captured on an Olympus FV1000 at random locations (Olympus, Richmond Hill, ON, CA).

Cell death assay: After treating HUVEC with sEVs (SEC-isolated) for 24 h, cells were fixed and permeabilised followed by staining with the In Situ Cell Death Kit (Sigma-Aldrich, Oakville, ON, Canada), according to the manufacturer's recommendations. Treatment with 2 U/ml of DNaseI (Thermo Fisher Scientific, Waltham, MA, USA) for 10 minutes at RT was used as a positive control for the assay.

Transcytosis assay: Total internal reflection fluorescence (TIRF) microscopy was performed on a Leica DMi8 microscope (Leica, Wetzlar, DE) with 63x/1.47 (O) objectives, 405 nm, 488 nm, 561 nm*,* and 637 nm laser lines, 450/50, 525/50, 600/50, 610/75 and 700/75 emission filters and run

with Quorum acquisition software (Quorum, Calgary, AB, CA). Microscope settings were kept constant between conditions. Image analysis was performed using a custom MATLAB single particle-tracking algorithm as previously reported [12]. Human Lung Microvascular Endothelial Cells (HMVEC-L, Lonza, Basel, CH) at 100% confluency were treated with 30 µl sEVs (in 1000 µl media) for one hour placed in a live cell imaging chamber and incubated with 10 $\mu g/ml^{-1}$ of AF488-Albumin (Thermo Fisher Scientific, Waltham, MA, USA) in cold HPMI media (Thermo Fisher Scientific, Waltham, MA, USA) for 10 minutes at 4°C to allow apical membrane-binding. Cells were starved for two hours before the treatment. Following membrane binding, cells were washed twice with ice-cold $PBS^{+/+}$ (Gibco, Gaithersburg, MD, USA) to remove unbound ligand. Following this, RT HPMI was added, and the cells were incubated on the live-cell imaging stage at 37°C for two minutes before initial image acquisition. Confluent regions of the monolayer were selected by viewing the number of nuclei in the DAPI field of view after staining with NucBlue Live ReadyProbes Reagent (Thermo Fisher Scientific, Waltham, MA, USA) and TIRF microscopy of the basal membrane performed to visualise exocytosis; each coverslip had 10-15 videos of 150 frames (100 ms exposure) captured.

Live imaging of actin dynamics: HUVEC were electroporated with LifeAct-GFP (Ibidi, Fitchburg, WI, USA) using nucleofection as before [13]. Cells (~30,000) were plated in each well of the two-well culture insert (Ibidi, Fitchburg, WI, USA). After removal of the insert, 500 µl of media was added and live imaging at 37°C in a 5% CO_2 humidified incubator using a Yokagawa Spinning Disk Confocal (Tokyo, Japan) was performed for 45-mintes. sEVs (45 µl, isolated by SEC) from diabetic or non-diabetic participant plasma were added and imaging was continued. For video quantification, cell boundaries were traced at ~3-min intervals over 35 minutes after the addition of sEVs. Cell traces for each cell were overlayed to visualise cell membrane fluctuations over time. Membrane displacement was quantified by taking the distance between the innermost trace and the outermost trace at various locations along the entire perimeter of a cell (minimum 32 measurements per cell). Membrane displacement measurements were normalised to the average membrane displacement of the non-diabetic sEV treatment group.

Statistical Analysis and Data Visualisation:

Randomisation and blinding procedures: Experimenter blinding of certain *in vitro* experiments was performed at the stage of assay initiation where possible (i.e., permeability, transcytosis, immunoblotting). However, experimenter blinding of *in vivo* studies was not performed due to stark phenotypic differences between control (*db/+*) and experimental (*db/db*) animals at the point of analysis. Nonetheless, where appropriate, analyses were conducted with masked assignment of groups to ensure reproducibility. Images presented in the manuscript and associated supplemental information were randomly selected images representative of original data from multiple experiments and, where used, serve merely as a visual representation of cumulative data collected. Power calculations were not performed to determine sample size as they were determined based on similar publications in the field. No datapoints were excluded from the analysis.

ESM References:

[1] Iyengar SK, Sedor JR, Freedman BI, et al. (2015) Genome-wide association and trans-ethnic meta-analysis for advanced diabetic kidney disease: Family Investigation of Nephropathy and Diabetes (FIND). PLoS genetics 11(8): e1005352. 10.1371/journal.pgen.1005352.
[2] Lacroix R, Judicone C, Mooberry M, Boucekine M, Key NS, Dignat-George F (2013) Standardization of pre-analytical variables in plasma microparticle determination: results of the International Society on Thrombosis and Haemostasis SSC Collaborative workshop. Journal of thrombosis and haemostasis 2013 Apr 2:10.1111/jth.12207. doi: 10.1111/jth.12207.
[3] Lucien F, Gustafson D, Lenassi M, et al. (2023) MIBlood-EV: Minimal information to enhance the quality and reproducibility of blood extracellular vesicle research. J Extracell Vesicles 12(12): e12385. 10.1002/jev2.12385
[4] Osteikoetxea X, Balogh A, Szabó-Taylor K, et al. (2015) Improved characterization of EV preparations based on protein to lipid ratio and lipid properties. PLoS One 10(3):e0121184. doi: 10.1371/journal.pone.0121184.
[5] Jeppesen DK, Hvam ML, Primdahl-Bengtson B, et al. (2014) Comparative analysis of discrete exosome fractions obtained by differential centrifugation. Journal of Extracellular Vesicles 3(1): 25011. 10.3402/jev.v3.25011
[6] (2001) BioCarta. Biotech Software & Internet Report 2(3): 117-120. 10.1089/152791601750294344
[7] Kanehisa M, Goto S (2000) KEGG: kyoto encyclopedia of genes and genomes. Nucleic Acids Res 28(1): 27-30. 10.1093/nar/28.1.27
[8] Jassal B, Matthews L, Viteri G, et al. (2020) The reactome pathway knowledgebase. Nucleic Acids Res 48(D1): D498-d503. 10.1093/nar/gkz1031
[9] Cheng HS, Besla R, Li A, et al. (2017) Paradoxical suppression of atherosclerosis in the absence of microRNA-146a. Circulation research 121(4): 354-367. 10.1161/CIRCRESAHA.116.310529.
[10] Shelke GV, Lässer C, Gho YS, Lötvall J (2014) Importance of exosome depletion protocols to eliminate functional and RNA-containing extracellular vesicles from fetal bovine serum. Journal of extracellular vesicles 3: 10.3402/jev.v3403.24783. 10.3402/jev.v3.24783
[11] DiStefano PV, Kuebel JM, Sarelius IH, Glading AJ (2014) KRIT1 Protein Depletion Modifies Endothelial Cell Behavior via Increased Vascular Endothelial Growth Factor (VEGF) Signaling. Journal of Biological Chemistry 289(47): 33054-33065. 10.1074/jbc.M114.582304
[12] Kraehling JR, Chidlow JH, Rajagopal C, et al. (2016) Genome-wide RNAi screen reveals ALK1 mediates LDL uptake and transcytosis in endothelial cells. Nature Communications 7(1): 13516. 10.1038/ncomms13516
[13] Nikolaev SI, Vetiska S, Bonilla X, et al. (2018) Somatic Activating KRAS Mutations in Arteriovenous Malformations of the Brain. N Engl J Med 378(3): 250-261. 10.1056/NEJMoa1709449

ESM TABLES:

ESM Table 1. Major resources table

REAGENT or RESOURCE	SOURCE/LOCATION	IDENTIFER
Western Blot and Immunofluorescence Antibodies		
Anti-Mouse-Alexa Flour 488	Thermo Fisher Scientific (Waltham, MA, USA)	Cat#: A-11059; RRID: AB_2534106
Goat Anti-Rabbit IgG HRP	Cell Signalling Technology (Danvers, MA, USA)	Cat#: 7074S; RRID: AB_2099233
Goat Anti-Rabbit-Alexa Flour 488	Thermo Fisher Scientific (Waltham, MA, USA)	Cat#: A11008; RRID: AB_143165
Horse Anti-Mouse IgG HRP	Cell Signalling Technology (Danvers, MA, USA)	Cat#: 7076; RRID: AB_330924
Mouse Anti-Human ALIX	Santa Cruz Biotechnology (Dallas, TX, USA)	Cat#: Sc-53540; RRID: AB_673819
Mouse Anti-Human CD63	Santa Cruz Biotechnology (Dallas, TX, USA)	Cat#: Sc-5275; RRID:AB_627877
Mouse Anti-Human GAPDH	Santa Cruz Biotechnology (Dallas, TX, USA)	Cat#: Sc-47724; RRID:AB_627678
Mouse Anti-Human VE-Cadherin	R&D Systems (Minneapolis, MN, USA)	Cat#: MAB9381; RRID: AB_2260374
Mouse Anti-Human VE-Cadherin	R&D Systems (Minneapolis, MN, USA)	Cat#: MAB9381; RRID:AB_2260374
Rabbit Anti-Human NFκB p65	Cell Signalling Technology (Danvers, MA, USA)	Cat#: D14E12; RRID: AB_2797709
Rabbit Anti-Human pAKT	Cell Signalling Technology (Danvers, MA, USA)	Cat#: 9272; RRID:AB_329827
Rabbit Anti-Human Phospho-AKT (Ser473)	Cell Signalling Technology (Danvers, MA, USA)	Cat#: 4060; RRID:AB_2315049
Rabbit Anti-Human Phospho-Myosin Light Chain 2	Cell Signalling Technology (Danvers, MA, USA)	Cat#: 3674S; RRID: AB_2147464
Rabbit Anti-Human Phospho-p44/42 (Thr202/Tyr204) MAP Kinase 1/2	Cell Signalling Technology (Danvers, MA, USA)	Cat#: 9101S; RRID: AB_331646
Rabbit Anti-Human VE-Cadherin	Cell Signalling Technology (Danvers, MA, USA)	Cat#: 2500; RRID:AB_10839118
Rabbit Anti-Mouse Claudin-5	Thermo Fisher Scientific (Waltham, MA, USA)	Cat#: 34-1600; RRID: AB_2533157
Rabbit Anti-mouse IgG-AlexaFluor 488	Thermo Fisher Scientific (Waltham, MA, USA)	Cat#: A11059; RRID:AB_2534106
Rabbit Anti-p44/42 MAP Kinase (Erk 1/2)	Cell Signalling Technology (Danvers, MA, USA)	Cat#: 4695S; RRID: AB_390779
Chemicals, Peptides, and Recombinant Proteins		
Round Cover Slip (8 mm)	VWR (Radnor, PA, USA)	Cat#: 101413-530
8M Urea	Sigma-Aldrich (Oakville, ON, Canada)	Cat#: U4883
Acetone	Sigma-Aldrich (Oakville, ON, Canada)	Cat#: 179124-500ML
Acrylamide	Sigma-Aldrich (Oakville, ON, Canada)	Cat#: A0924
AF488-Albumin	Thermo Fisher (Scientific (Waltham, MA, USA)	Cat#: A13100

Agarose	Sigma-Aldrich (Oakville, ON, Canada)	Cat#: A4718
Ammonium Bicarbonate	Sigma-Aldrich (Oakville, ON, Canada)	Cat#: 09830
Bio-Safe Coomassie Stain	Bio-Rad (Philadelphia, PA, USA)	Cat#: 161-0786
Blotting Grade Blocker Non-Fat Milk	Bio-Rad (Philadelphia, PA, USA)	Cat#: 1706404XTU
Bovine Serum Albumin	BioShop (Burlington, ON, Canada)	Cat#: ALBC0100
Collagen Type I, Rat Tail	Sigma-Aldrich (Oakville, ON, Canada)	Cat#: 08-115
Dimethyl Sulfoxide	Sigma-Aldrich (Oakville, ON, Canada)	Cat#: D8418
Dithiothreitol	Sigma-Aldrich (Oakville, ON, Canada)	Cat#: 43816
Endothelial Cell Medium Complete	ScienCell (Carlsbad, CA, USA)	Cat#: 1001
Fibronectin	Sigma-Aldrich (Oakville, ON, Canada)	Cat#: 10838039001
FITC-10 Kilodalton Dextran	Thermo Fisher Scientific (Waltham, MA, USA)	Cat#: D1860
FITC-40 Kilodalton Dextran	Sigma-Aldrich (Oakville, ON, Canada)	Cat#: 53379
Formic Acid (0.1% LC-MS Grade)	Thermo Fisher Scientific (Waltham, MA, USA)	Cat#: 85170
Hanks Buffered Salt Solution	Thermo Fisher Scientific (Waltham, MA, USA)	Cat#: 14065-056
Hydrochloric Acid	Sigma-Aldrich (Oakville, ON, Canada)	Cat#: 320331
Horseradish Peroxidase	Sigma-Aldrich (Oakville, ON, Canada)	Cat#: P8375-2KU
Human Plasma-Like Medium	Thermo Fisher Scientific (Waltham, MA, USA)	Cat#: A4899101
Hydrochloric Acid	Sigma-Aldrich (Oakville, ON, Canada)	Cat#: 320331-2.5L
Isoflurane	Dechra (Pointe-Claire, QC, Canada)	Cat#: N/A
EDTA	Thermo Fisher Scientific (Waltham, MA, USA)	Cat#: 15575020
Laemmli Sample Buffer (4x)	Bio-Rad (Philadelphia, PA, USA)	Cat#: 1610747
Latex Transfer Standards (100 nm*)*	Malvern Panalytical (Amesbury, UK)	Cat#: NTA4088
Latex Transfer Standards (200 nm*)*	Malvern Panalytical (Amesbury, UK)	Cat#: NTA4089
LC-MS Grade Water	Thermo Fisher Scientific (Waltham, MA, USA)	Cat#: 51140
Mounting Medium with DAPI	Vector Laboratories (Burlingame, CA, USA)	Cat#: H-1200
MultiScribe™ Reverse Transcriptase	Thermo Fisher Scientific/Applied Biosystems (Waltham, MA, USA)	Cat#: 4311235
Paraformaldehyde	Sigma-Aldrich/Millipore (Oakville, ON, Canada)	Cat#: P6148-1KG

Phosphate Buffered Saline	Thermo Fisher Scientific/Gibco (Waltham, MA, USA)	Cat#: LS10010023
PhosStop Tablets	Roche (Mississauga, ON, Canada)	Cat#: 04906845001
Pierce™ Luciferase Cell Lysis Buffer	Thermo Fisher Scientific (Waltham, MA, USA)	Cat#: 16189
Ponceau S	Thermo Fisher Scientific (Waltham, MA, USA)	Cat#: S2872
Protease Inhibitor Tablets	Roche (Mississauga, ON, Canada)	Cat#: 11836170001
ProteaseMAX™ Surfactant	Promega (San Luis Obispo, CA, USA)	Cat#: V2071
Protein Standard	GeneDirex (Taoyuan City, Taiwan)	Cat#: PM007-0500
Proteinase K	Thermo Fisher Scientific (Waltham, MA, USA)	Cat#: EO0491
Rat Tail Collagen I	Sigma-Aldrich (Oakville, ON, Canada)	Cat#: C3867
Rhodamine-Phalloidin	Thermo Fisher Scientific (Waltham, MA, USA)	Cat#: R415
RIPA Buffer (10x)	EMD Millipore (Burlington, MA, USA)	Cat#: 20-188
RNasin Ribonuclease Inhibitor	Promega (San Luis Obispo, CA, USA)	Cat#: N2111
SL327	Sigma-Aldrich (Oakville, ON, Canada)	Cat#: S4069
Streptavidin Sepharose	Cell Signalling Technology (Danvers, MA, USA)	Cat#: 3419
Sulfo-NHS-Biotin	BioVision/Abcam (Boston, MA, USA)	Cat#: 2322-50
Texas Red-70 Kilodalton	Thermo Fisher Scientific (Waltham, MA, USA)	Cat#: D1864
TMB Substrate	Cell Signalling Technology (Danvers, MA, USA)	Cat#: 7004P4
TNFα Human	Sigma-Aldrich (Oakville, ON, Canada)	Cat#: T0157-10UG
Tris-Buffer Saline	Thermo Fisher Scientific (Waltham, MA, USA)	Cat#: J60764.K2
Triton X-100	Sigma-Aldrich (Oakville, ON, Canada)	Cat#: T8787
Trypsin/Lys-C	Promega (San Luis Obispo, CA, USA)	Cat#: V5073
Tween	Sigma-Aldrich (Oakville, ON, Canada)	Cat#: P1379
UltraPure Glycine	Thermo Fisher Scientific (Waltham, MA, USA)	Cat#: 15527013
Vascular Endothelial Growth Factor	Sigma-Aldrich (Oakville, ON, Canada)	Cat#: V4512-10UG
Vybrant DyeCycle Orange Stain	Thermo Fisher Scientific (Waltham, MA, USA)	Cat#: V35005
Y-27632 Dihydrochloride	MedChemExpress (Monmouth Junction, NJ, USA)	Cat#: HY-10583
Commercial Components and Assays		
60 mm Tissue Culture Plates	Sigma-Aldrich/Corning (New York, NY, USA)	Cat#: 430166

Acclaim Pepmap Easy Spray	Thermo Fisher Scientific (Waltham, MA, USA)	Cat#: ES75150PN
Amersham™ Hybond®	Sigma-Aldrich/GE Healthcare (Oakville, ON, Canada)	Cat#: GE10600023
Amicon Ultra-15 Centrifugal Filters	Sigma-Aldrich/Millipore Sigma (Oakville, ON, Canada)	Cat#: UFC901024
Black 96 Well Assay Plate	Sigma-Aldrich (Oakville, ON, Canada)	Cat#: M0312
Bovine Serum Albumin	ScienCell (Carlsbad, CA, USA)	Cat#: A3294
Costar TC-Treated Plates (24-well)	Sigma-Aldrich/Millipore Sigma (Oakville, ON, Canada)	Cat#: CLS3527-100EA
Coverslips No. 1, 24x50 mm	VWR (Radnor, PA, USA)	Cat#: 4839081
LoBind Tubes	Eppendorf (Mississauga, ON, Canada)	Cat#: 22431021 / 022431081
DNase I Solution	ThermoFisher Scientific (Waltham, MA, USA)	Cat#: 89836
Dual Luciferase Reporter Assay	Promega (San Luis Obispo, CA, USA)	Cat#: 16186
Dulbecco's Modified Eagle's Medium	Thermo Fisher Scientific (Waltham, MA, USA)	Cat#: 12491015
Dulbecco's Phosphate Buffered Saline	Sigma-Aldrich (Oakville, ON, Canada)	Cat#: D8537
ExoQuick	System Biosciences (Palo Alto, CA, USA)	Cat#: EXOQ20A-1
Fetal Bovine Serum	Thermo Fisher Scientific (Waltham, MA, USA)	Cat#: 16000
Gelatin	Thermo Fisher Scientific (Waltham, MA, USA)	Cat#: AC611995000
In Situ Cell Death Kit	Sigma-Aldrich (Oakville, ON, Canada)	Cat#: 11684795910
Iodoacetic Acid	Sigma-Aldrich (Oakville, ON, Canada)	Cat#: I4386
K_2EDTA Lavender Hemogard	BD Biosciences (San Jose, CA, USA	Cat#: 02-657-32
LifeAct-GFP	Ibidi (Fitchburg, WI, USA)	Cat#: 60112
Lipofectamine 2000	Thermo Fisher Scientific (Waltham, MA, USA)	Cat#: 11668019
Low Concentration BCA	Abcam (Boston, MA, USA)	Cat#: ab207002
Luciferase Construct	Promega (San Luis Obispo, CA, USA)	Cat#: N/A
Mini-PROTEAN TGX 4-20%	Bio-Rad (Philadelphia, PA, USA)	Cat#: 4561096
Mycoplasma Detection Kit	Lonza (Basel, CH)	Cat#: LT07-318
Nuc Lab-Tek II Chamber Slide	Thermo Fisher Scientific (Waltham, MA, USA)	Cat#: 154534
NucBlue Live ReadyProbes Reagent	Thermo Fisher Scientific (Waltham, MA, USA)	Cat#: R37606
Passive Lysis Buffer	Promega (San Luis Obispo, CA, USA)	Cat#: E1941
Phosphate Buffered Saline $^{+/+}$	Gibco (Gaithersburg, MD, USA)	Cat#: 10010023
Penicillin-Streptomycin	ScienCell (Carlsbad, CA, USA)	Cat#: 0513
Phosphopeptide Enrichment Kit	Thermo Fisher Scientific (Waltham, MA, USA)	Cat#: A32992

pRL Renilla	Promega (San Luis Obispo, CA, USA)	Cat#: E2231
qEV Single – 70 nm (1st generation)	IZON Science (Medford, MA, USA)	Cat#: SP2
Quantifoil R 2/2 Copper Grids	Electron Microscopy (Hatfield, PA, USA)	Cat#: Q210CR2
RNeasy Mini Kit	Qiagen (Germantown, MD, USA)	Cat#: 74104
Super Signal Femto	Thermo Fisher Scientific (Waltham, MA, USA)	Cat#: 34094
SuperSignal West Pico PLUS™	Thermo Fisher Scientific (Waltham, MA, USA)	Cat#: 34580
Syringe (23-gauge)	BD Biosciences (San Jose, CA, USA)	Ca#: Z192449
Syringe (29-gauge)	BD Biosciences (San Jose, CA, USA)	Cat#: 305930
Syringe Filter (0.05 µm)	Sigma-Aldrich/Millipore Sigma (Oakville, ON, Canada)	Cat#: VMWP02500
Syringe Filter (0.22 µm)	Sigma-Aldrich/Millipore Sigma (Oakville, ON, Canada)	Cat#: GSWP04700
Transwell Filters (12-well plate)	Corning Life Sciences (New York, NY, USA)	Cat#: 3462
Two-Well Culture Insert	Ibidi (Fitchburg, WI, USA)	Cat#: 80209
Whatman™ Quantitative Filter Paper	Thermo Fisher Scientific (Mississauga, ON, Canada)	Cat#: 09-927-541
Cell Lines		
b.END3	ATCC (Manassas, VA, USA)	Cat#: CRL-2299
hCMEC/D3	Cellutions Biosystems (Toronto, ON, CA)	Cat#: CLU512
Human Lung Microvascular ECs	Lonza (Basel, CH)	Cat#: CC-2527
Pooled HUVEC	Cedarlane/PromoCell (Burlington, ON, Canada)	Cat#: C-12203
Instruments / Tools		
ChemiDoc	Bio-Rad (Philadelphia, PA, USA)	Cat#: 12003153
Cytation5	BioTek (Winooski, VT, USA)	Cat#: BTCYT5FAV
Dionex Ultimate 3000 uHPLC	Thermo Fisher Scientific (Waltham, MA, USA)	Cat#: N/A
DS-11+ Spectrophotometer	DeNovix (Wilmington, DE, United States)	Cat#: DS-11+
Ear Bars	Narishige (Tokyo, Japan)	Cat#: EB-3B
GloMax Luminometer	Promega (San Luis Obispo, CA, USA)	Cat#: E5311
High-Speed Micro-Drill	Harvard Apparatus (Holliston, MA, USA)	Cat#: 75-1887
Leica DMi8 Microscope	Leica (Wetzlar, DE)	Cat#: 8121493
NanoSight NS300	Malvern Panalytical (Amesbury, UK)	Cat#: N/A
Olympus FV1000	Olympus (Richmond Hill, ON, CA)	Cat#: FV1000
Roche 480 Instrument II Lightcycler	Roche (Mississauga, ON, Canada)	Cat#: N/A

Rodent Warming Pad	Thermo Fisher Scientific (Waltham, MA, USA)	Cat#: 50-195-4000
Spinning Disk Confocal	Yokagawa (Tokyo, Japan)	Cat#: CSU-W1
Stereotaxic Frame	Narishige (Tokyo, Japan)	Cat#: SR-5M-HT
Talos L120C	Thermo Fisher Scientific (Waltham, MA, USA)	Cat#: L120C
Thermo Orbitrap Fusion	Thermo Fisher Scientific (Waltham, MA, USA)	Cat#: IQLAAEGAAPFADBMBHQ
Two-Photon Microscope	Leica (Wetzlar, DE)	Cat#: N/A
Vitrobot	Field Electron and Ion (Eindhoven, NL)	Cat#: N/A
xCELLigence RTCA DP	Agilent (Santa Clara, CA, USA)	Cat#: 05228972001
Murine Models and Husbandry		
BKS.Cg-Dock7$^{m/+}$ +/+ Lepr^{db}/J; db/+	The Jackson Laboratory (Bar Harbour, MI, USA)	Cat#: 000642
BKS.Cg-Lepr^{db} /+ Lepr^{db}/J; db/db	The Jackson Laboratory (Bar Harbour, MI, USA)	Cat#: 000642
C57BL/6J	The Jackson Laboratory (Bar Harbour, MI, USA)	Cat#: 000664
Standard Chow	Research Diets (New Brunswick, NJ, USA)	Cat#: D12450B
Software		
Consensus PathPB	http://cpdb.molgen.mpg.de/YCPDB	
Fiji-ImageJ	https://imagej.net/software/fiji/	
Illustrator	https://www.adobe.com/products/illustrator	
Mascot	https://www.matrixscience.com/	
MATLAB	https://www.mathworks.com/products/matlab.html	
Prism 9.0	https://www.graphpad.com/features	
Quorum Acquisition Software	https://www.volocity4d.com/	
Scaffold	https://www.proteomesoftware.com/products/scaffold-5	

ESM Table 2. Nanoparticle tracking software settings

NANOSIGHT SETTINGS	SETTING LEVEL
Capture	
Laser type	Blue 488 nm
Camera level	13
Slide shutter	1232
Slider gain	219
Shutter/ms	30.8
Camera histogram upper limit	9000
Camera histogram lower limit	80
Frame rate/fps	25.0
Analysis	
Detection threshold	3
Maximum jump mode	Auto
Blur	Auto
Minimum track length	Auto
Total frames analysed	1498

ESM Table 3. Polymerase chain reaction primers

PAIR NUMBER	NAME	DIRECTION	SEQUENCE
1	*IL-6*	Forward	CTGGCAGAAAACAACCTGAA
1	*IL-6*	Reverse	ACCAGGCAAGTCTCCTCATT
2	*IL-8*	Forward	AGGACAAGAGCCAGGAAGAA
2	*IL-8*	Reverse	TTTAGCACTCCTTGGCAAAA
3	*E-Selectin*	Forward	CTGGCCTGCTACCTACCTGT
3	*E-Selectin*	Reverse	AGCTACCAAGGGAATGTTGG
4	*VCAM-1*	Forward	GTTGAAGGATGCGGGAGTAT
4	*VCAM-1*	Reverse	GGATGCAAAATAGAGCACGA
5	*ICAM-1*	Forward	CGGCCAGCTTATACACAAGA
5	*ICAM-1*	Reverse	GTCTGCTGGGAATTTTCTGG
6	*CCL2*	Forward	TCATAGCAGCCACCTTCATT
6	*CCL2*	Reverse	CGAGCCTCTGCACTGAGAT
7	*IL-1β*	Forward	ACCTCCAGGGACAGGATATG
7	*IL-1β*	Reverse	AACACGCAGGACAGGTACAG
8	*GAPDH*	Forward	AGGTGAAGGTCGGAGTCAAC
8	*GAPDH*	Reverse	GAGGTCAATGAAGGGGTCAT

ESM Table 4. Differentially expressed proteins in murine small extracellular vesicles

**Identified Proteins**	**Accession**	***p* value**	**Fold Change***	**Profile**
Upregulated (*db/db*)				
α-1B-glycoprotein	A1BG_MOUSE	0.014	INF	*db/db* high, *db/+* low
β-2-glycoprotein	APOH_MOUSE	0.026	5	*db/db* high, *db/+* low
Complement C4-B	CO4B_MOUSE	0.0075	1.4	*db/db* high, *db/+* low
Fibronectin	FINC_MOUSE	0.0029	1.7	*db/db* high, *db/+* low
Ig α chain C region	IGHA_MOUSE	0.038	1.7	*db/db* high, *db/+* low
Ig κ chain V-V region L7	KV5A9_MOUSE	0.047	1.3	*db/db* high, *db/+* low
Immunoglobulin heavy constant μ	IGHM_MOUSE	0.022	1.3	*db/db* high, *db/+* low
Integrin α-M	ITAM_MOUSE	0.031	INF	*db/db* high, *db/+* low
Mannan-binding lectin serine protease 1	MASP1_MOUSE	0.045	1.4	*db/db* high, *db/+* low
Mannan-binding lectin serine protease 2	MASP2_MOUSE	0.026	1.7	*db/db* high, *db/+* low
Pregnancy zone protein	PZP_MOUSE	0.019	1.7	*db/db* high, *db/+* low
Prosaposin	SAP_MOUSE	0.0075	2.5	*db/db* high, *db/+* low
Proteasome subunit β type-4	PSB4_MOUSE	0.039	2.5	*db/db* high, *db/+* low
Proteasome subunit β type-6	PSB6_MOUSE	0.035	2.5	*db/db* high, *db/+* low
Protein AMBP	AMBP_MOUSE	0.02	2.5	*db/db* high, *db/+* low
Secreted phosphoprotein 24	SPP24_MOUSE	0.02	10	*db/db* high, *db/+* low
Serum amyloid A-4 protein	SAA4_MOUSE	0.016	1.4	*db/db* high, *db/+* low
Vitronectin	VTNC_MOUSE	0.03	2	*db/db* high, *db/+* low
Downregulated (Db/Db)				
Actin, cytoplasmic 1	ACTB_BOVIN	0.043	0.4	*db/db* low, *db/+* high
Complement C1q subcomponent subunit A	C1QA_MOUSE	0.033	0.7	*db/db* low, *db/+* high
Beta-lactoglobulin	LACB_BOVIN	0.025	0.2	*db/db* low, *db/+* high
Carboxylesterase 3A	EST3A_MOUSE	0.016	0.4	*db/db* low, *db/+* high
Structural maintenance of chromosomes protein	SMC1B_MOUSE	0.016	0	*db/db* low, *db/+* high
Complement C1q TNF-related protein 3	C1QT3_MOUSE	0.025	0	*db/db* low, *db/+* high

Abbreviations: AMBP = α-1-microglobulin/bikunin precursor; *db/db* = *db/db* mouse sEVs; *db/+* = *db/+* mouse sEVs; Ig = Immunoglobulin; INF = infinite; TNF = Tumor Necrosis Factor
* Fold-change is protein abundance in *db/db* sEVs compared to *db/+* sEVs. A fold change of 'INF' indicates that the protein was not detected in *db/+* sEVs and a fold change of '0' indicates that the protein was not detected in *db/db* sEVs.

ESM Table 5. Differentially expressed proteins in human small extracellular vesicles

Identified Proteins	Accession	*p* value	Fold Change*	Profile
Upregulated (Diabetic)				
10 kDa heat shock protein, mitochondrial	P61604	0.016	3.3	Non-diabetic low, diabetic high
26S proteasome regulatory subunit 7	P35998	0.00045	INF	Non-diabetic low, diabetic high
Aflatoxin B1 aldehyde reductase member 2	O43488	0.00045	INF	Non-diabetic low, diabetic high
α-1B-glycoprotein	P04217	0.033	INF	Non-diabetic low, diabetic high
Amyloid-β A4 protein	P05067 (+5)	0.011	10	Non-diabetic low, diabetic high
Annexin A4	P09525 (+1)	0.00045	INF	Non-diabetic low, diabetic high
C4b-binding protein α chain	P04003	0.041	2	Non-diabetic low, diabetic high
Calpain-1 catalytic subunit	P07384	0.0048	INF	Non-diabetic low, diabetic high
Cathepsin D	P07339	0.031	INF	Non-diabetic low, diabetic high
Caveolae-associated protein 2	O95810	0.039	5	Non-diabetic low, diabetic high
Cofilin-1	P23528	0.0041	5	Non-diabetic low, diabetic high
Complement component C8 β chain	P07358	0.00045	INF	Non-diabetic low, diabetic high
Cytosol aminopeptidase	P28838 (+1)	0.00045	INF	Non-diabetic low, diabetic high
Desmoplakin	P15924	0.00045	INF	Non-diabetic low, diabetic high
Dolichyl-diphosphooligosaccharide-protein glycosyltransferase subunit 1	P04843	0.00045	INF	Non-diabetic low, diabetic high
Endothelial cell-selective adhesion molecule	Q96AP7	0.00045	INF	Non-diabetic low, diabetic high
Eukaryotic initiation factor 4A-I	P60842	0.00045	INF	Non-diabetic low, diabetic high
Fructose-bisphosphate aldolase A	P04075	0.007	3.3	Non-diabetic low, diabetic high
Genome polyprotein	P13901	0.00045	INF	Non-diabetic low, diabetic high
Glucose-6-phosphate isomerase	P06744 (+1)	0.0063	INF	Non-diabetic low, diabetic high
Glyceraldehyde-3-phosphate dehydrogenase	P04406	0.02	11.1	Non-diabetic low, diabetic high
Growth factor receptor-bound protein 2	P62993	0.00045	INF	Non-diabetic low, diabetic high
Guanine nucleotide-binding protein G(I)/G(S)/G(O) subunit γ-11	P61952	0.00045	INF	Non-diabetic low, diabetic high
Guanine nucleotide-binding protein G(I)/G(S)/G(T) subunit β-2	P62879	0.00045	INF	Non-diabetic low, diabetic high
Guanine nucleotide-binding protein subunit α-13	Q14344	0.00045	INF	Non-diabetic low, diabetic high
Hemoglobin subunit β	P68871	0.019	3.3	Non-diabetic low, diabetic high
Immunoglobulin heavy constant α-1	P01876	0.039	2.5	Non-diabetic low, diabetic high
Immunoglobulin λ variable 10-54	A0A075B6I4	0.00045	INF	Non-diabetic low, diabetic high
Importin-7	O95373	0.00045	INF	Non-diabetic low, diabetic high
Integrin α-2	P17301	0.0018	2.5	Non-diabetic low, diabetic high
Isoform 2 of ADP-ribosylation factor 3	P61204-2 (+2)	0.00045	INF	Non-diabetic low, diabetic high
Isoform 2 of Dynamin-1-like protein	O00429-3	0.017	10	Non-diabetic low, diabetic high
Isoform 2 of Guanine nucleotide-binding protein G(I)/G(S)/G(T) subunit β-1	P62873-2	0.031	INF	Non-diabetic low, diabetic high
L-lactate dehydrogenase A chain	P00338 (+1)	0.008	INF	Non-diabetic low, diabetic high
Macrophage migration inhibitory factor	P14174	0.00045	INF	Non-diabetic low, diabetic high
Major prion protein	P04156 (+2)	0.00045	INF	Non-diabetic low, diabetic high
Malate dehydrogenase, cytoplasmic	P40925 (+2)	0.00045	INF	Non-diabetic low, diabetic high
Microtubule-associated protein RP/EB family member 2	Q15555 (+3)	0.027	10	Non-diabetic low, diabetic high

Myosin light chain kinase, smooth muscle	Q15746 (+6)	0.00045	INF	Non-diabetic low, diabetic high
Nucleoside diphosphate kinase B	P22392 (+1)	0.017	INF	Non-diabetic low, diabetic high
Osteoclast-stimulating factor 1	Q92882	0.00045	INF	Non-diabetic low, diabetic high
Peptidyl-prolyl cis-trans isomerase FKBP2	P26885	0.00045	INF	Non-diabetic low, diabetic high
Peroxiredoxin-5, mitochondrial	P30044 (+1)	0.00045	INF	Non-diabetic low, diabetic high
Phosphoglucomutase-1	P36871 (+1)	0.00045	INF	Non-diabetic low, diabetic high
Prolactin-inducible protein	P12273	0.00045	INF	Non-diabetic low, diabetic high
Proteasome subunit β type-1	P20618	0.00045	INF	Non-diabetic low, diabetic high
Protein kinase C and casein kinase substrate in neurons protein 2	Q9UNF0 (+1)	0.00045	INF	Non-diabetic low, diabetic high
Rab GDP dissociation inhibitor α	P31150 (+1)	0.045	INF	Non-diabetic low, diabetic high
Rab GDP dissociation inhibitor β	P50395	0.026	INF	Non-diabetic low, diabetic high
Ras-related protein Ral-B	P11234 (+2)	0.00045	INF	Non-diabetic low, diabetic high
Receptor-type tyrosine-protein phosphatase eta	Q12913	0.00045	INF	Non-diabetic low, diabetic high
Regulator of G-protein signalling 10	O43665 (+2)	0.00045	INF	Non-diabetic low, diabetic high
Rho GDP-dissociation inhibitor 2	P52566	0.049	INF	Non-diabetic low, diabetic high
Rho GTPase-activating protein 18	Q8N392 (+1)	0.00045	INF	Non-diabetic low, diabetic high
Septin-7	Q16181 (+1)	0.029	3.3	Non-diabetic low, diabetic high
SH3 domain-binding glutamic acid-rich-like protein 2	Q9UJC5	0.00045	INF	Non-diabetic low, diabetic high
Small membrane A-kinase anchor protein	Q9BSF0	0.00045	INF	Non-diabetic low, diabetic high
Small ubiquitin-related modifier 3	P55854 (+4)	0.00045	INF	Non-diabetic low, diabetic high
Stress-induced-phosphoprotein 1	P31948 (+2)	0.00045	INF	Non-diabetic low, diabetic high
Superoxide dismutase [Mn], mitochondrial	P04179 (+1)	0.00045	INF	Non-diabetic low, diabetic high
T-complex protein 1 subunit **ε**	P48643	0.00045	INF	Non-diabetic low, diabetic high
Tenascin	P24821 (+1)	0.00045	INF	Non-diabetic low, diabetic high
Tetraspanin-14	Q8NG11 (+2)	0.00045	INF	Non-diabetic low, diabetic high
Translationally-controlled tumor protein	P13693	0.00045	INF	Non-diabetic low, diabetic high
Tripeptidyl-peptidase 2	P29144	0.00045	INF	Non-diabetic low, diabetic high
Ubiquitin carboxyl-terminal hydrolase 5	P45974 (+1)	0.00045	INF	Non-diabetic low, diabetic high
Vinculin	P18206 (+1)	0.0071	10	Non-diabetic low, diabetic high
Vitamin K-dependent protein S	P07225	0.035	1.27	Non-diabetic low, diabetic high
Wiskott-Aldrich syndrome protein	P42768	0.00045	INF	Non-diabetic low, diabetic high
Downregulated (Diabetic)				
Apolipoprotein A-II	P02652	0.027	0.5	Non-diabetic high, diabetic low
Isoform 4 of Clusterin	P10909-4	0.0012	0.5	Non-diabetic high, diabetic low
Protein S100-A4	P26447	0.026	0	Non-diabetic high, diabetic low
Trypsin-1	P07477	0.0014	0	Non-diabetic high, diabetic low
Apolipoprotein C-I	P02654	0.0014	0	Non-diabetic high, diabetic low
HLA class I histocompatibility antigen, A-34 α chain	P30453	0.0014	0	Non-diabetic high, diabetic low
Secretory carrier-associated membrane protein 2	O15127	0.0014	0	Non-diabetic high, diabetic low
Immunoglobulin λ constant 7	A0M8Q6	0.024	0	Non-diabetic high, diabetic low

* Fold-change is protein abundance in Diabetic sEVs compared to Non-diabetic sEVs. INF = infinite. A fold change of 'INF' indicates that the protein was not detected in Non-diabetic sEVs and a fold change of '0' indicates that the protein was not detected in Diabetic sEVs.

ESM FIGURES AND LEGENDS:

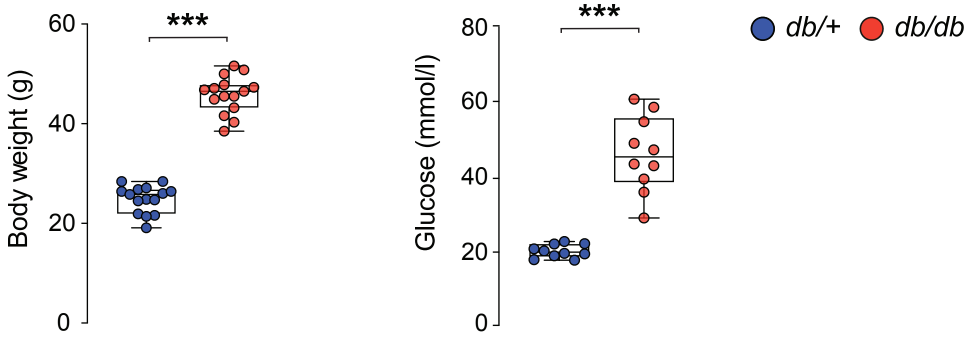

ESM Figure 1: *Db/db* mice recapitulate a type II diabetes mellitus phenotype. Body-weight measurements and blood glucose measurements of *db/+* and *db/db* mice at 14-weeks of age ($p<0.001$; n=15 and n=10 biological replicates, respectively).

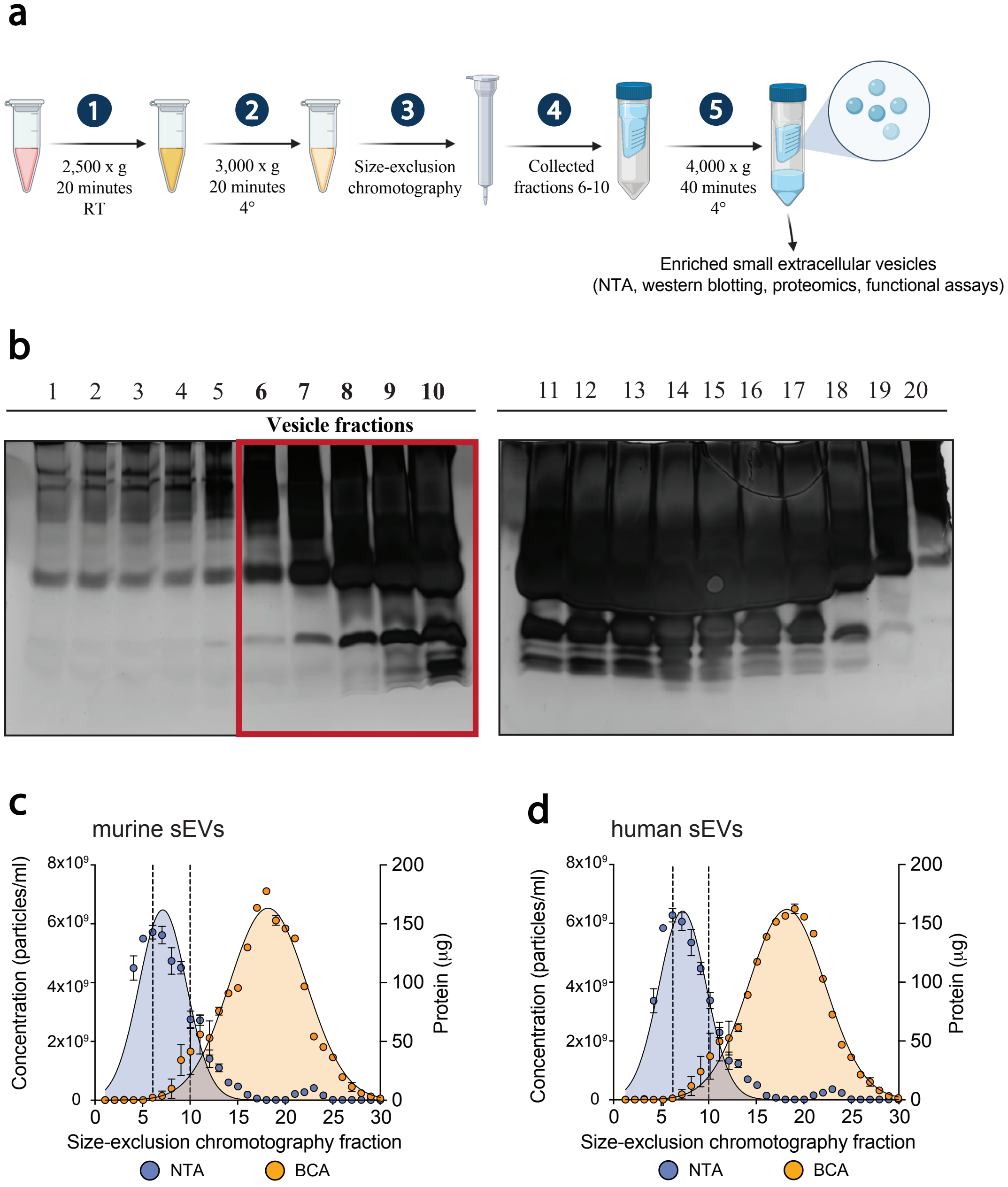

ESM Figure 2: Size-exclusion chromatography effectively enriches small extracellular vesicles from plasma. (a) Schematic detailing the size-exclusion chromatography (SEC) protocol for the enrichment of sEVs from plasma. **(b)** SEC fractions from human plasma (1-20) were resolved on a 4-20% SDS-PAGE gel and stained for total protein content using silver stain. Fractions enriched in sEVs (6-10) displayed lower total concentrations of protein compared to later fractions (11-20). **(c, d)** Overlay of particle concentrations determined by Nanoparticle Tracking Analysis (NTA) with protein concentrations across all SEC fractions for murine and human plasma samples, respectively. Values are represented as the mean ± S.D. (n=3 biological replicates). Fractions 6-10 have high particle to protein ratios (see Figure 1c). Abbreviations: BCA =

Bicinchoninic acid; NTA = Nanoparticle Tracking Analysis; SDS-PAGE = Sodium Dodecyl-Sulfate Polyacrylamide Gel Electrophoresis; sEVs = Small Extracellular Vesicles.

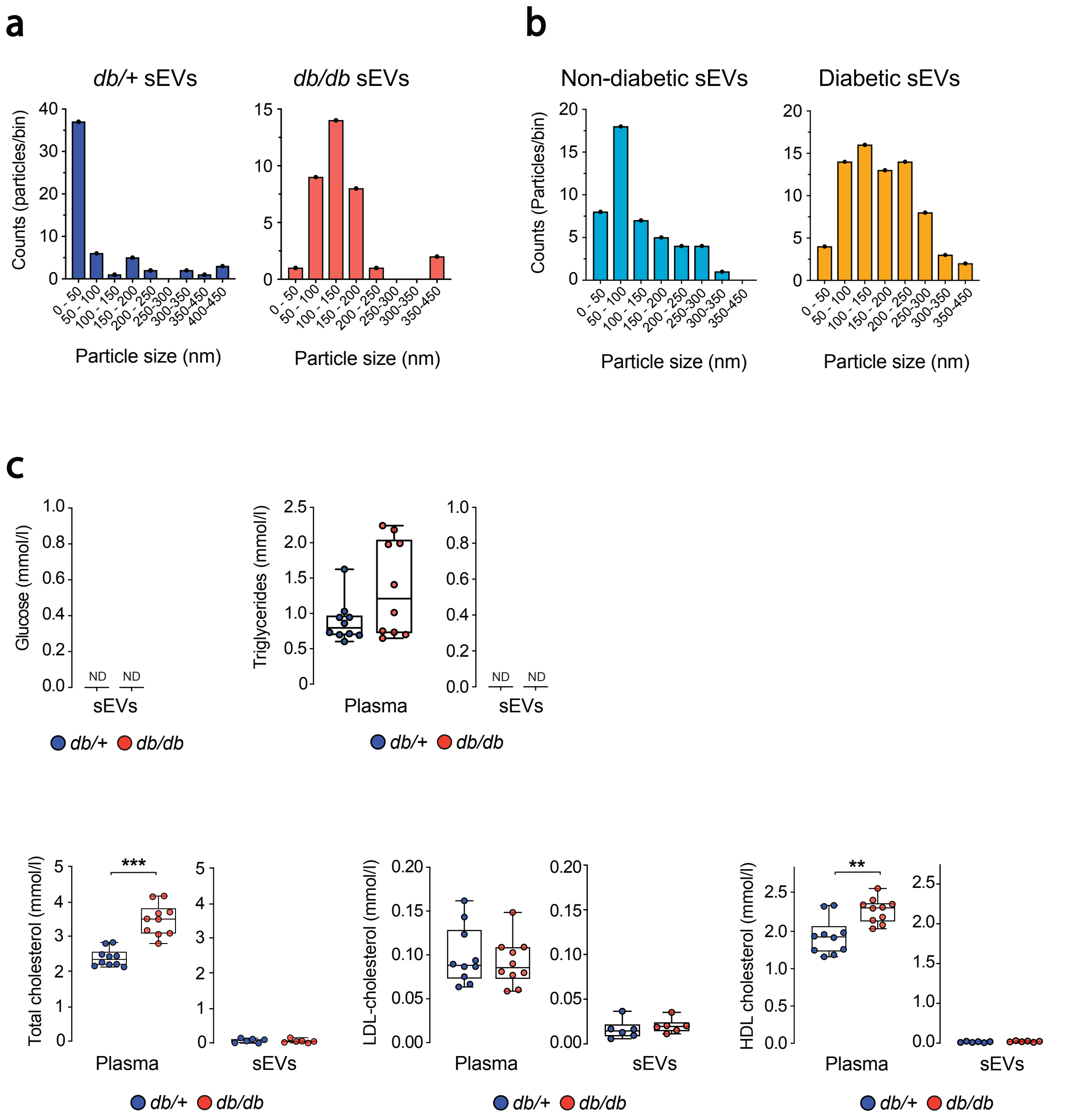

ESM Figure 3: Small extracellular vesicles enriched by size-exclusion chromatography do not contain significant lipoprotein or glucose contamination. (a, b) Quantification of small extracellular vesicle (sEV) size and raw counts from CryoTEM images of murine and human plasma, respectively (see Fig. 1a for representative images). Bar graphs represent a summation of particle sizes estimated from randomly selected 500 nm scale cryoEM images (n=3 biological replicates). **(c)** Quantification of glucose, triglycerides, LDL-cholesterol, total cholesterol and HDL-cholesterol in plasma (n=10 biological replicates) and corresponding SEC-enriched sEVs (n=6 biological replicates) from *db/+* and *db/db* mice. Glucose and triglycerides were not detected (ND), while total cholesterol, LDL cholesterol and HDL cholesterol were near the level of detection; plasma glucose levels are shown in ESM Figure 1. The data were analysed using

unpaired t-tests and asterisks indicate comparisons that are significantly different (*p < 0.05, **p < 0.01, ***p < 0.001); values are represented as the mean ± min and max. Abbreviations: CryoEM = Cryogenic Electron Microscopy; HDL = High-Density; Lipoprotein; LDL = Low-Density Lipoprotein; ND = Not Detected; sEVs = Small Extracellular Vesicles.

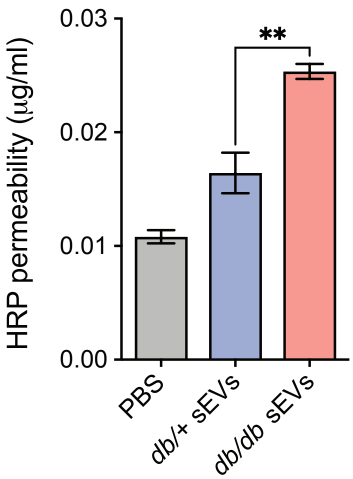

ESM Figure 4: Equalising for EV number still results in enhanced leakage from diabetic small extracellular vesicles. Nanoparticle tracking analysis (NTA) was used to quantify the concentration of sEVs from *db/db* and *db/+* mice and an equal number of vesicles (10^{10}) were added to confluent HUVEC transwells for 24 hours, after which permeability to horseradish peroxidase (HRP) was measured. sEVs isolated from diabetic mice induced more leak than an equal number of vesicles from non-diabetic mice. The data represents technical triplicate measurements from a representative experiment. The data were analysed using an unpaired t-test and the asterisk (**) indicates $p < 0.01$. Abbreviations: HRP = horseradish peroxidase; NTA = Nanoparticle tracking analysis.

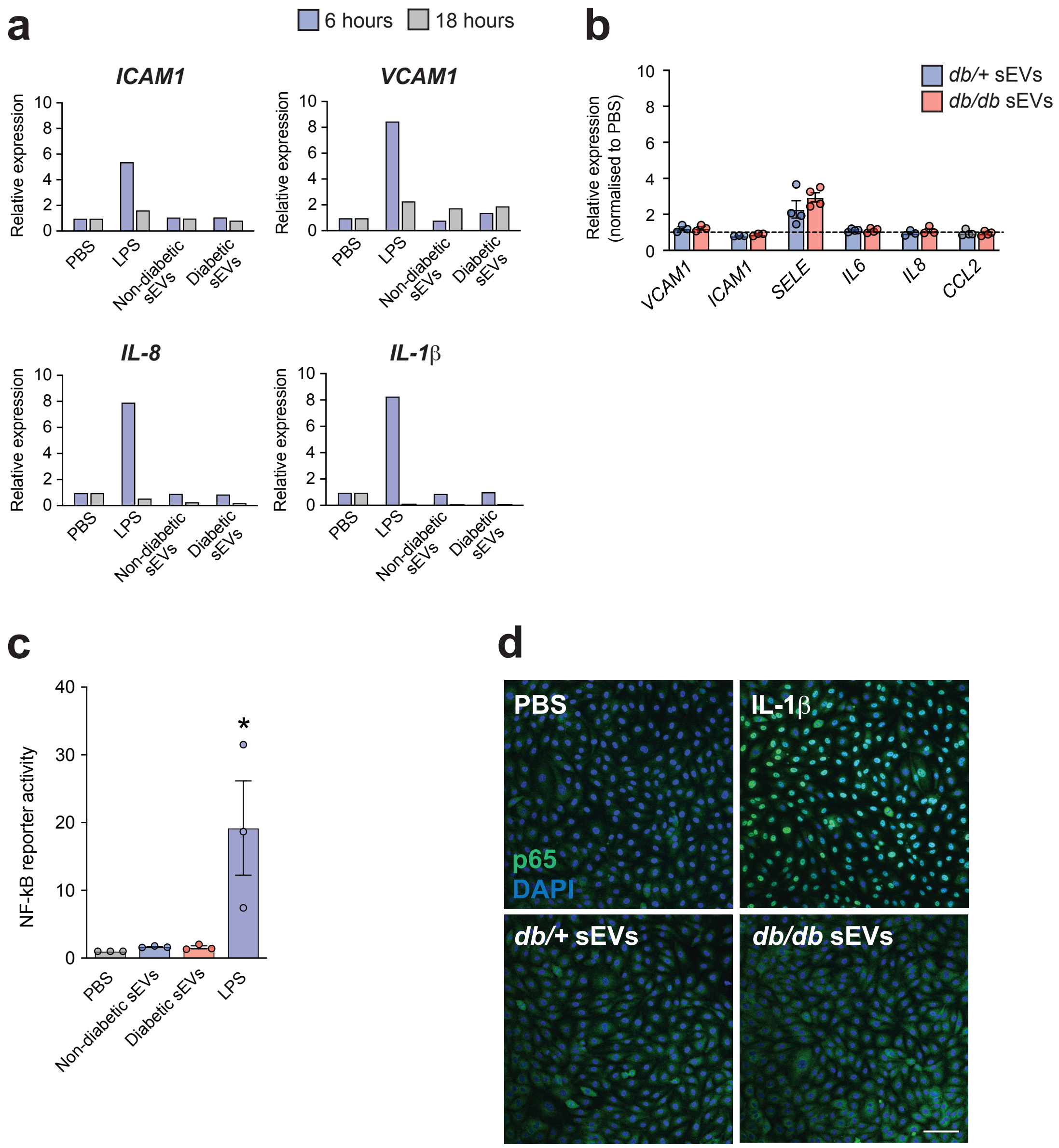

ESM Figure 5: Small extracellular vesicles do not differentially induce inflammation. (a) HUVEC were treated with SEC-isolated human plasma sEVs for 6 or 18 hours and the expression of inflammatory genes was assessed by qRT-PCR. Lipopolysaccharide was added as a positive control for inflammatory signalling. A representative experiment is shown. **(b)** HUVEC were treated with ExoQuick-isolated plasma sEVs (*db/+* or *db/db*) for 24 hours and inflammatory genes were assessed by qRT-PCR (n=3-4 biological replicates per group). **(c)** NF-κB reporter activity was assessed in hCMEC/D3 ECs following treatment with SEC-isolated plasma EVs (*db/+* or *db/db*) for 6 hours (n=3 biological replicates per group). **(d)** Nuclear localisation of p65 was visualised in HUVEC treated with SEC-isolated plasma EVs from non-diabetic or diabetic individuals for two hours. IL-1β was added as a positive control for inflammatory signalling. Scale bar = 100 μm. Abbreviations: hCMEC/D3 = human cerebral endothelial cell line D3; HUVEC =

Human Umbilical Vein Endothelial Cells; IL-1β = Interleukin 1 Beta; SEC = Size-Exclusion Chromatography; qRT-PCR = Real-Time Quantitative Reverse Transcription PCR; sEVs = Small Extracellular Vesicles.

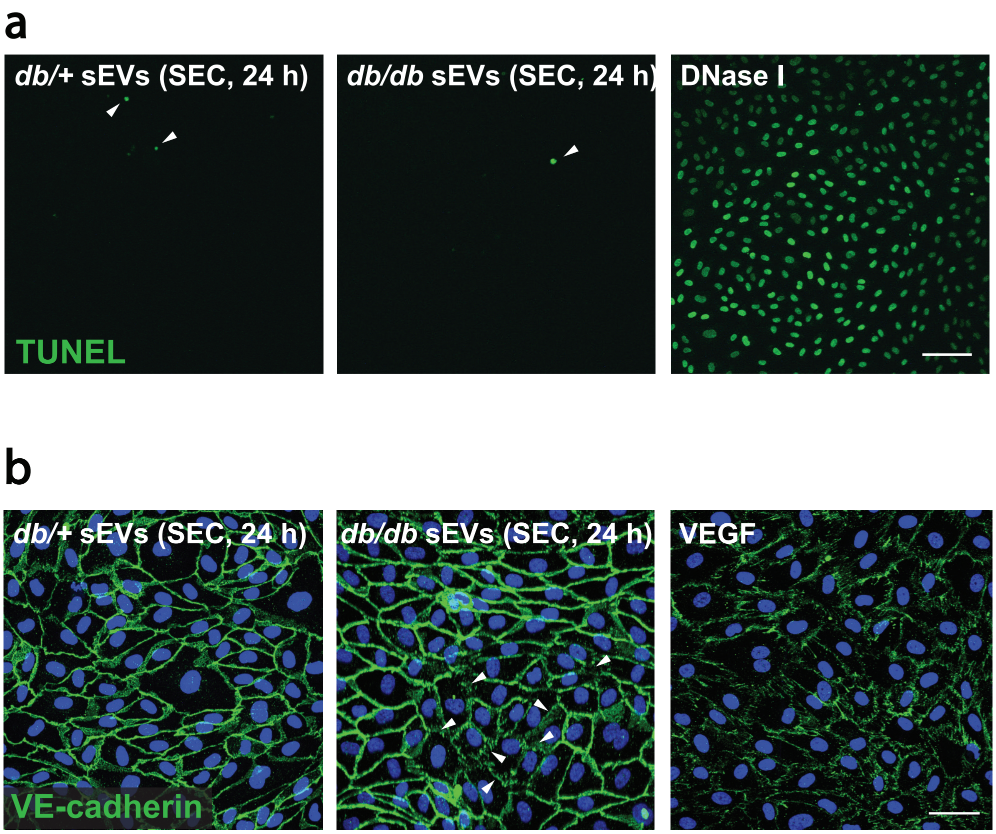

ESM Figure 6: Diabetic small extracellular vesicles disrupt adherens junctions but do not induce cell death. (a) TUNEL staining was performed on human umbilical vein endothelial cells (HUVEC) treated with *db/+* or *db/db* sEVs for 24 hours. Treatment with DNase I was included as a positive control for the TUNEL assay. A representative experiment is shown. Scale bar = 100 µm. **(b)** HUVEC were treated for 24 hours with small extracellular vesicles (sEVs) enriched by size exclusion chromatography (SEC) from *db/+* or *db/db* plasma and adherens junctions were visualised by vascular endothelial (VE)-Cadherin staining. Vascular endothelial growth factor (VEGF) was added as a positive control. Arrowheads indicate regions of adherens junction disassembly. Representative images are shown. Scale bar = 50 µm. Abbreviations: HUVEC = Human Umbilical Vein Endothelial Cells; SEC = Size-Exclusion Chromatography; sEVs = Small Extracellular Vesicles; VE-Cadherin = Vascular Endothelial-Cadherin; VEGF = Vascular endothelial growth factor.

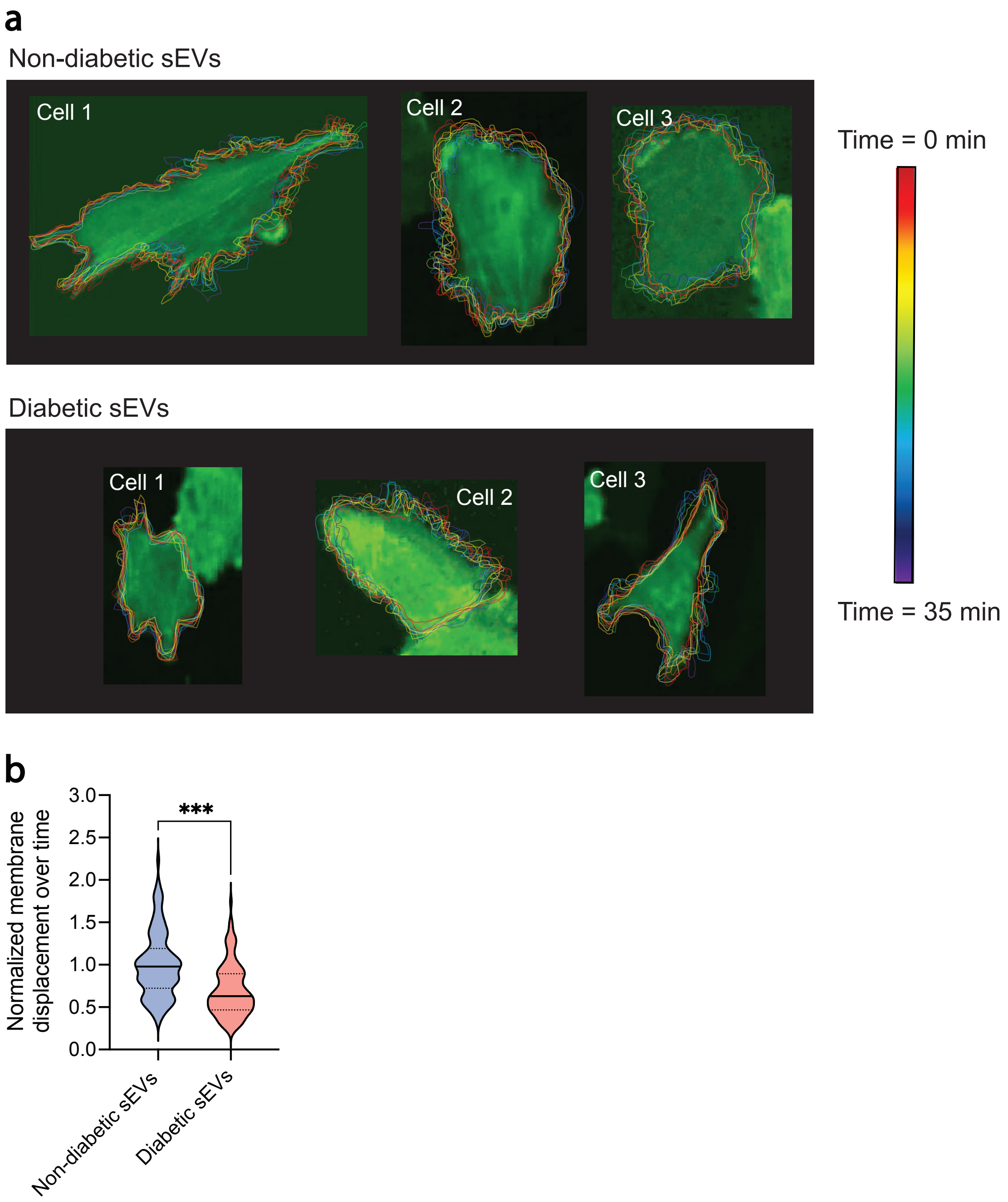

ESM Figure 7: Small extracellular vesicles isolated from diabetic human plasma reduces membrane displacement. (a) Representative cell images from time-lapse microscopy are shown. HUVEC cells were electroporated with LifeAct-GFP constructs (green) to visualise actin cytoskeleton. Cell boundary traces were overlayed onto each image and were colour-coded from time 0-minute (purple) to time 35-minute (red) of the time-lapse video. **(b)** Quantification of the time-lapse imaging experiment showing normalised membrane displacement over time for HUVEC cells treated with diabetic and non-diabetic sEVs. A minimum of 32 measurements were made around the perimeter of each cell. Mann-Whitney test was used for statistical analysis. n=3

cells per group. *** p-value < 0.001. Abbreviations: HUVEC = Human Umbilical Vein Endothelial Cells; sEVs = Small Extracellular Vesicles.
